# Lhx1 Is Required for Specification of the Renal Progenitor Cell Field

**DOI:** 10.1371/journal.pone.0018858

**Published:** 2011-04-15

**Authors:** M. Cecilia Cirio, Zhao Hui, Caroline E. Haldin, Chiara Cianciolo Cosentino, Carsten Stuckenholz, Xiongfong Chen, Sung-Kook Hong, Igor B. Dawid, Neil A. Hukriede

**Affiliations:** 1 Department of Developmental Biology, University of Pittsburgh, Pittsburgh, Pennsylvania, United States of America; 2 School of Biomedical Sciences, The Chinese University of Hong Kong, Ma Liu Shui, Hong Kong; 3 Department of Medicine, University of Pittsburgh, Pittsburgh, Pennsylvania, United States of America; 4 Unit on Biologic Computation, Eunice Kennedy Shriver National Institute of Child Health and Human Development, National Institutes of Health (NIH), Bethesda, Maryland, United States of America; 5 Laboratory of Molecular Genetics, Eunice Kennedy Shriver National Institute of Child Health and Human Development, National Institutes of Health (NIH), Bethesda, Maryland, United States of America; 6 Drug Discovery Institute, University of Pittsburgh, Pittsburgh, Pennsylvania, United States of America; King's College London, United Kingdom

## Abstract

In the vertebrate embryo, the kidney is derived from the intermediate mesoderm. The LIM-class homeobox transcription factor *lhx1* is expressed early in the intermediate mesoderm and is one of the first genes to be expressed in the nephric mesenchyme. In this study, we investigated the role of Lhx1 in specification of the kidney field by either overexpressing or depleting *lhx1* in *Xenopus* embryos or depleting *lhx1* in an explant culture system. By overexpressing a constitutively-active form of Lhx1, we established its capacity to expand the kidney field during the specification stage of kidney organogenesis. In addition, the ability of Lhx1 to expand the kidney field diminishes as kidney organogenesis transitions to the morphogenesis stage. In a complimentary set of experiments, we determined that embryos depleted of *lhx1*, show an almost complete loss of the kidney field. Using an explant culture system to induce kidney tissue, we confirmed that expression of genes from both proximal and distal kidney structures is affected by the absence of *lhx1*. Taken together our results demonstrate an essential role for Lhx1 in driving specification of the entire kidney field from the intermediate mesoderm.

## Introduction

The vertebrate kidney performs an essential function of removing waste products from the blood and osmoregulation. Although functionally similar, three types of kidneys have evolved in vertebrates, the pronephros, mesonephros and metanephros, with specification of each kidney induced by the preceding nephric tissue. This inductive event results in a progressively more complex organization ending with the metanephric kidney, which exists in mammals and birds [1], [2], [3]. All three kidneys share the same basic structure and organization of the functional unit, the nephron, but differ mostly in the number and spatial assembly of nephrons. Each nephron consists of the renal corpuscle and the renal tubule [4]. In amphibians the pronephric kidney is the functional embryonic kidney. In *Xenopus laevis*, it consists of a bilaterally paired organ, comprising a single non-integrated nephron on each side of the embryo [5].

The entire kidney field is derived from the intermediate mesoderm, which lies between the paraxial and lateral plate mesoderm. Within the intermediate mesoderm, specification of renal progenitor cells follows a precise temporal and spatial sequence of signaling events [6], [7]. Tissues juxtaposed to the intermediate mesoderm have been shown to influence patterning of the kidney field. Embryological experiments in chicken revealed that initial patterning of the kidney field is dependent upon signals from the axial/paraxial mesoderm [2], [3], [8], [9], with retinoic acid (RA) emanating from the paraxial mesoderm required for the initial specification of renal progenitor cells [7], [10], [11]. Recent studies have demonstrated that ectopic expression of RA increases the size of the kidney field, while blocking the pathway inhibits specification [10], [12]. In *Xenopus*, embryos treated with RA show increased expression of early kidney markers and treatment of *Xenopus* pluripotent explants with a combination of RA and Activin induces most kidney cell types [10], [13]. In addition, bone morphogenetic proteins (BMP) originating from the lateral plate mesoderm also influence kidney specification. Intermediate mesoderm fate commitment is regulated by a dose-dependent activation of the BMP signaling cascade along the embryonic dorso-ventral axis [2], [14]. Low levels of BMP activate intermediate mesoderm gene expression, whereas high levels of BMP repress intermediate mesoderm gene expression and activates lateral plate mesoderm genes [14].

During embryogenesis, processes such as body axis determination, as well as tissue and regional specification, require the participation of the LIM homeodomain family of transcription factors [15]. The LIM homeodomain transcription factors contain two cysteine-histidine rich motifs (LIM domains), a central homeodomain and a C-terminal transactivation domain [16]. The LIM domains are thought to function as protein interaction modules that can regulate the function of different components in a transcriptional complex [15]. The LIM homeodomain transcription factor Lhx1 (formerly known as Xlim1 in *Xenopus laevis*), interacts with the LIM binding protein, Ldb, and this interaction triggers Lhx1 activation [17], [18]. *Lhx1* is initially expressed in the Spemann-Mangold organizer in *Xenopus* [19], a region that coordinates cell fate specification and axis formation [20], [21]. In mouse and *Xenopus* embryos, *lhx1* is required for proper cell movements during gastrulation [22]. In addition, hyperactive forms of Lhx1 have been shown to induce axis duplication in *Xenopus* embryos [23]. Taken together, these findings indicate a conserved role of Lhx1 in early embryonic patterning.

*Lhx1* is one of the earliest genes to be expressed in the pronephric anlagen [24], [25], [26], [27], [28]. In *Xenopus*, expression of *lhx1* in the lateral plate mesoderm and intermediate mesoderm is initially seen by stage 12.5, starts to condense into a stripe of intermediate mesoderm between stages 15–18, converges to the nephric field at around stage 19, and finally is expressed in the presumptive nephrostomes and tubule at stage 29/30 [25], [29]. When a dominant-negative form of *lhx1* is expressed in the anterior kidney field, expression of proximal tubule markers is lost [30]. Coexpression of *lhx1* and *pax8* results in the development of enlarged kidney and the formation of ectopic pronephric tubules [25]. In addition, *lhx1* expression has been shown to be an early molecular marker of the forming zebrafish mesonephros and the first molecular marker of renal progenitor cells during adult zebrafish nephrogenesis [31]. Lhx1 also plays an important role at multiple stages of mammalian kidney development. In the mouse, *lhx1* is expressed early in the intermediate mesoderm [24], [32] and is required for the correct patterning of the kidney field [33]. Later in the developing metanephros, Lhx1 is required for ureteric bud morphogenesis and patterning of the nephric vesicle [34], [35]. Finally, in *Xenopus* embryos, downregulation of *lhx1* is required for proper differentiation of the pronephric kidney. Persistent *lhx1* expression in *miR-30a-5p* depleted embryos results in normal kidney field specification, but in a failure of kidney cells to terminally differentiate [36].

In the present report, we address the involvement of Lhx1 in events that control specification of renal progenitor cells from the intermediate mesoderm. We approach this question by studying the development of the presumptive pronephros in embryos in which *lhx1* is either overexpressed or depleted and show that pronephric kidney formation is drastically affected. In addition, by overexpressing a constitutively-active form of Lhx1 in a temporally-controlled manner, we establish that this transcription factor can expand the nephric field during the kidney specification stage [37], [38]. Finally, by using an *in vitro* explant culture system and microarray analysis we demonstrate that loss of *lhx1* results in lack of expression of markers from all the domains of the kidney. Taken together, the data suggest that *lhx1* expression is necessary for the early patterning the of entire kidney field.

## Results

### Over-expression of a constitutively-active form of Lhx1 expands the kidney field

*Lhx1* and *pax8* are expressed early in the pronephric anlagen (**Fig. S1**) and coexpression of these two genes has a synergistic effect that results in the development of an enlarged kidney and/or the formation of ectopic pronephric tubules [25], [39]. For a more detailed study of *lhx1* over-expression in *Xenopus* pronephric kidney formation without *pax8* coexpression [25], we used two constitutively-active forms of Lhx1 [23]. In *Xenopus*, *lhx1* is highly expressed in the organizer region of the early gastrula embryo, and perturbation of endogenous expression in this region causes gastrulation defects [19], [22], [23]. To lessen the effect of these gastrulation defects, we targeted the ventral region of the embryo. Specifically, we injected one of the V2 blastomeres (1xV2) of 8-cell embryos since a preponderance of the kidney tissue has been shown by fate mapping to be derived from the V2 blastomeres [40].

**Figure 1:**
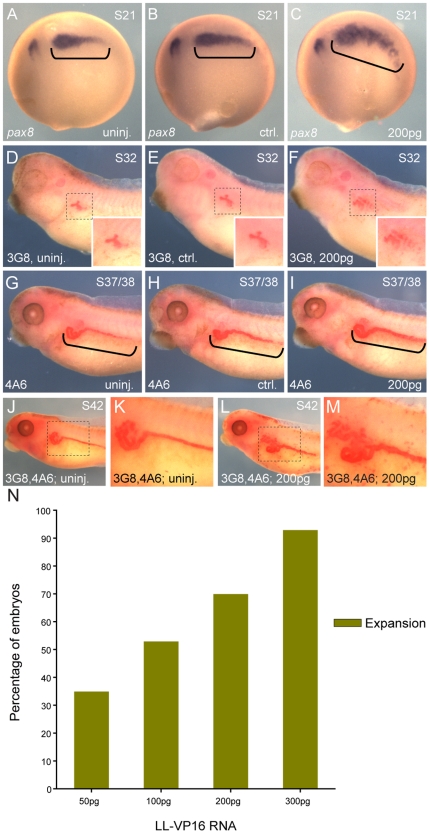
Over-expression of *lhx1* induces expansion of the pronephric kidney. Embryos were injected (1xV2) with 200 pg of LL-VP16 mRNA at the 8-cell stage. (**A–C**) *In situ* hybridization of embryos at stage 20 for the early pronephric marker *pax8*. (**A**) Uninjected embryo. (**B–C**) Control and injected sides of the same embryo are shown. (**C**) Expansion of *pax8* expression was observed in 83% of the embryos (n = 33). Kidney fields are highlighted with black brackets. (**D–I**) 3G8 and 4A6 whole-mount immunostaining were carried out at stages 32 and 37/38, respectively. (**D, G**) Uninjected embryos. (**E, F, H, I**) Control and injected sides of the same embryo. (**F, I**) Larger tubule epithelium with 3G8 was observed in 81% of the embryos (n = 32) and expanded intermediate and distal tubules with 4A6 were observed in 80% of the embryos (n = 30). (**D–F**) Insets show enlargements of 3G8 staining of the proximal tubule. (**J–M**) 3G8, 4A6 double whole-mount immunostaining of stage 42 embryos. (**K**) Magnification of the pronephric kidney of uninjected embryo. (**M**) Magnification of the pronephric kidney of injected embryo. (**N**) Bar graph showing the percentage of embryos injected at the different doses of LL-VP16 that showed expansion of *pax8* expression. LL-VP16 mRNA 50 pg: expansion of *pax8* expression was observed in 35% of the embryos (n = 26). LL-VP16 mRNA 100 pg: expansion of *pax8* expression was observed in 53% of the embryos (n = 30). LL-VP16 mRNA 200 pg: expansion of *pax8* expression was observed in 70% of the embryos (n = 30). LL-VP16 mRNA 300 pg: expansion of *pax8* expression was observed in 93% of the embryos (n = 27).

We tested the constitutively-active constructs for their ability to induce expansion of the pronephric anlage as assessed by *pax8 in situ* hybridization at stage 20 (**Fig. S2, S3**). The constructs are fusions of Lim domain binding protein 1 (Ldb1) and Lhx1, which had been shown to functionally replace Lhx1 in depletion and over-expression assays [23]. The Ldb1-Lhx1 constitutively-active construct (LL-CA) is a fusion of the Ldb1 dimerization domain with the linker, C-terminal and homeodomain of Lhx1, while the Ldb1-Lhx1-VP16 construct (LL-VP16) replaces the Lhx1 C-terminal domain with the transactivation domain of the viral protein VP16 [23]. Injection of 1200 pg of LL-CA mRNA (1xV2) induced expansion of the pronephric kidney in 82% of the injected embryos (**Fig. S2N**), while 200 pg of LL-VP16 mRNA induced expansion of the pronephric kidney in 70% of the injected embryos (**Fig. 1A–1C, 1N**). Based on these results, we decided to continue with the LL-VP16 construct since it allowed us to inject a lower dose of mRNA to achieve a similar effect. We limited the dose of LL-VP16 mRNA to 200 pg, since at higher doses injected embryos showed a misshapen kidney field (**Fig. S3L**).

To address how the expansion of the pronephric anlagen translates to the differentiated kidney field, we performed whole-mount immunostaining with two antibodies, 3G8 and 4A6, that recognize the proximal tubules or the early distal and distal tubules, respectively [41], [42]. Embryos injected with LL-VP16 mRNA and stained with 3G8 antibody showed a larger differentiated tubule epithelium (81%) (**Fig. 1D–1F**). Staining with 4A6 showed expanded early distal and distal tubules on the injected side of embryos (80%) (**Fig. 1G–1I**). Double immunostaining showed that both regions of the pronephros are enlarged on the side of the embryos injected with LL-VP16 mRNA, but the relative juxtaposition of each marker is not affected (**Fig. 1J–1M**). These results demonstrate that over-expression of constitutively-active Lhx1 induces an expansion of the kidney field, without overtly changing the patterning of the tubule segments.

### Lhx1-mediated expansion of the kidney field takes place during a restricted temporal window

**Figure 2:**
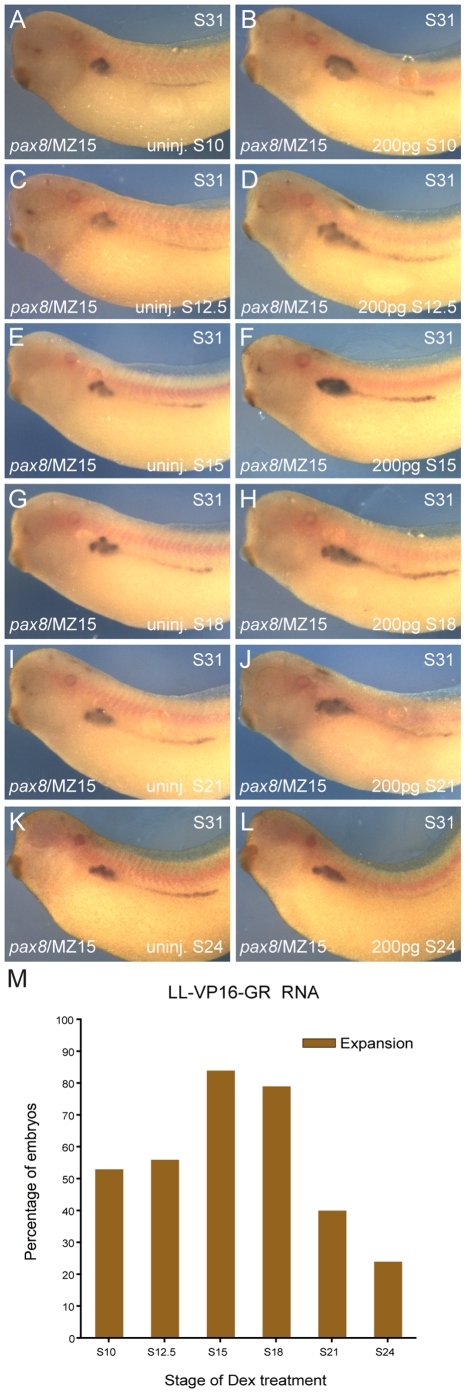
Inducible activation of Lhx1, defines a temporal window of kidney field expansion. Embryos were injected (1xV2) with 200 pg of LL-VP16-GR mRNA at the 8-cell stage. (**A–L**) *In situ* hybridization for *pax8* of embryos at stage 31, followed by MZ15 whole-mount immunostaining. (**A, C, E, G, I, K**) Uninjected embryos. (**B, D, F, H, J, L**) Injected embryos. Activation of LL-VP16-GR was controlled by addition of dexamethasone (Dex) at specified stages. Dex was added to uninjected and injected embryos at: (**A, B**) stage 10; (**C, D**) stage 12.5; (**E, F**) stage 15; (**G, H**) stage 18; (**I, J**) stage 21; (**K, L**) stage 24. (**M**) Bar graph with the percentage of injected embryos that showed expansion after Dex treatment at different stages. Expansion of *pax8* expression was observed in 53% of the embryos for stage 10 (n = 36), 56% for stage 12.5 (n = 32), 84% for stage 15 (n = 32), 79% for stage 18 (n = 34), 40% for stage 21 (n = 45) and 24% for stage 24 (n = 21).

In order to establish a more precise time frame in which Ldb1-Lhx1 functions during pronephric kidney development, we temporally-controlled LL-VP16 activation by fusing the glucocorticoid receptor (GR) ligand-binding domain to the 3′ end of the LL-VP16 construct (LL-VP16-GR). Addition of dexamethasone (Dex) results in nuclear translocation of the fusion protein and activation of LL-VP16-GR. Embryos were injected with 200 pg of LL-VP16-GR mRNA (1xV2) and Dex was added at different stages prior to or during pronephric kidney specification (stages 10, 12.5, 15, 18) and morphogenesis (stages 21 and 24) [37]. Injected and uninjected control embryos were fixed at stage 31 and processed for *in situ* hybridization of *pax8*. When Dex was added to injected embryos at stages 10 and 12.5, 53% and 56% of the embryos, respectively, showed an expansion of *pax8* expression (**Fig. 2A–2D, 2M**). When Dex was added later during specification (stages 15 and 18) a greater percentage of the embryos (84% and 79%, respectively) showed an expanded kidney field (**Fig. 2E–2H, 2M**). Only 40% and 24% of the embryos treated with Dex at stages 21 and 24, respectively, showed expansion (**Fig. 2I–2L, 2M**). To confirm possible axis duplication was not contributing to the expanded kidney field the embryos were immunostained for either the notochord marker MZ15, or the muscle-specific antibody 12/101 [25], [43]. Neither the MZ15 or 12/101 staining showed duplicate axes (**Fig. 2A–L** **, Fig. S4**). The efficacy of LL-VP16-GR to induce expansion of the pronephric kidney field was significantly reduced when kidney organogenesis transitions from the specification to morphogenesis stages [37]. Taken together, these data indicate that *Ldb1-Lhx1* transcriptional complex plays an important role in regulating the size of the kidney field during the specification stage, but this effect is reduced once the kidney field undergoes morphogenesis.

### *Lhx1* over-expression expands intermediate mesoderm progenitors at the expense of paraxial mesoderm

**Figure 3:**
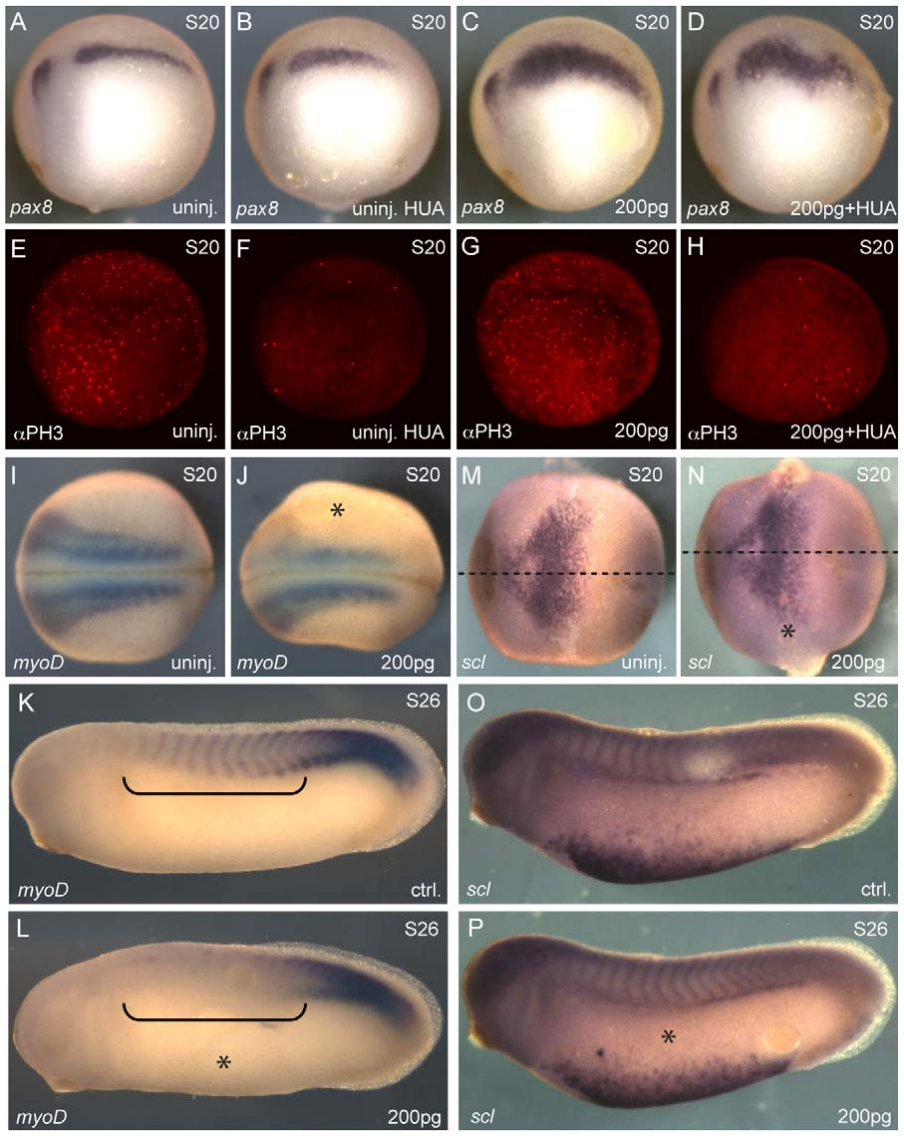
Ectopic *lhx1* expression causes a fate transformation event. Embryos were injected (1xV2) with 200 pg of LL-VP16 mRNA at the 8-cell stage. Embryos were treated with hydroxyurea and aphidicolin (HUA) at stage 10.5/11. (**A–D**) *In situ* hybridization of embryos at stage 20 for *pax8*. (**A**) Uninjected embryo. (**B**) Uninjected embryo treated with HUA. (**C**) Injected embryo. Expansion of *pax8* expression was observed in 91% of the embryos (n = 35). (**D**) Injected embryo treated with HUA. Expansion of *pax8* expression was observed in 82% of the embryos (n = 33). (**E–H**) Whole-mount immunostaining analysis of proliferation with anti-phospho-Histone H3 antibody, αPH3. (**E**) Uninjected embryo. (**F**) Uninjected embryo treated with HUA. (**G**) Injected embryo. (**H**) Injected embryo treated with HUA. (**I–L**) *In situ* hybridization of embryos for the paraxial mesoderm marker *myoD*. (**I, J**) Uninjected and injected embryos at stage 20. Reduced *myoD* expression was observed in 63% of the embryos (n = 51). (**K, L**) Control and injected sides of the same embryo at stage 26. Reduced *myoD* expression was observed in 87% of the embryos (n = 30). Anterior somites are highlighted with black brackets. (**M–P**) *In situ* hybridization of embryos for the lateral plate mesoderm marker *scl*. (**M, N**) Uninjected and injected embryos at stage 20 (n = 45). Midline of the embryos is marked with a dotted line with anterior to the left. (**O, P**) Control and injected sides of the same embryo at stage 26 (n = 43). The injected side of the embryos is marked with an asterisk.

To assess whether expansion of the pronephric kidney in embryos overexpressing LL-VP16 was due to increased proliferation of intermediate mesoderm progenitors or a fate transformation event, we treated LL-VP16 mRNA injected embryos (1xV2) at stage 10.5/11 with the DNA synthesis inhibitors hydroxyurea and aphidicolin (HUA), which have been shown to block cell division without affecting cell fate determination [44]. Embryos were fixed at stage 20 and assayed for changes in *pax8* expression. As compared to LL-VP16 mRNA injected embryos, embryos injected with LL-VP16 mRNA and treated with HUA showed a dramatic decrease in the number of mitotic cells, assessed by immunofluorescence with anti-phospho-Histone H3 antibody, but still displayed an expansion of the pronephric kidney field (91% compare to 82%) (**Fig. 3A–3H**). This suggests that an increase in cell proliferation is unlikely to be a major contributor to Ldb1-Lhx1 mediated expansion of the kidney field.

Coexpression of *lhx1* and *pax8* induces enlarged pronephroi and an associated reduction of somitic tissue [25]. Therefore, we wanted to determine if Lhx1 affects tissue juxtaposed to the intermediate mesoderm by using LL-VP16 over-expression. To investigate the possibility that cells from the paraxial mesoderm and/or lateral plate mesoderm have acquired intermediate mesoderm fate, we analyzed expression of the paraxial mesoderm marker *myoD* and the lateral plate mesoderm marker *scl* by *in situ* hybridization. We observed reduced *myoD* expression on the injected side of embryos at stage 20 (63%) (**Fig. 3I, 3J**) and at stage 26 (87%) (**Fig. 3K, 3L**). Expression of *scl* was not affected in embryos at either stage 20 (**Fig. 3M, 3N**) or stage 26 (**Fig. 3O, 3P** **)** injected with LL-VP16 RNA. These results suggest that Ldb1-Lhx1 transcriptional complex is able to induce fate transformation of cells from the paraxial mesoderm to contribute to the intermediate mesoderm and adopt a pronephric kidney fate.

### Depletion of *lhx1* using *lhx1-AS* results in a loss of the kidney field

**Figure 4:**
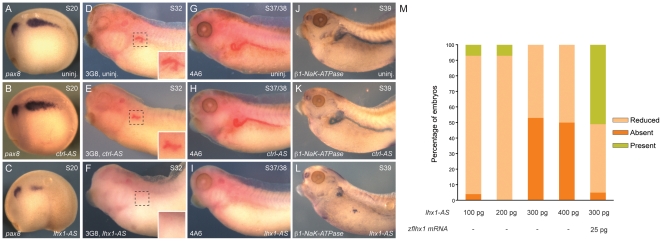
Depletion of *lhx1* results in a loss of the pronephric kidney. Embryos were injected (1xV2) with 300 pg of *ctrl-AS* or *lhx1-AS* at the 8-cell stage. (**A, D, G, J**) Uninjected embryos. (**B, E, H, K**) Embryos injected with *ctrl-AS*. (**C, F, I, L**) Embryos injected with *lhx1-AS*. (**A–C**) *In situ* hybridization of embryos at stage 20 for the pronephric marker *pax8*. (**C**) Reduced or absent *pax8* expression was observed in 47% and 53% of the embryos, respectively (n = 34). (**D–F**) 3G8 whole-mount immunostaining was carried out at stage 32. (**E**) Reduced 3G8 staining was observed in 5% of the embryos (n = 41). (**F**) Reduced 3G8 staining was observed in 76% of the embryos (n = 34). Insets show enlargement of 3G8 staining of the proximal tubule. (**G–I**) 4A6 whole-mount immunostaining was carried out at stage 37/38. (**H**) Reduced 4A6 staining was observed in 9% of the embryos (n = 23). (**I**) Reduced 4A6 staining was observed in 91% of the embryos (n = 34). (**J–L**) *In situ* hybridization of embryos at stage 39 for *β1-NaK-ATPase*. (**L**) Reduced *β1-NaK-ATPase* expression was observed in 72% of the embryos (n = 29). (**M**) Bar graph with the percentage embryos injected with the different doses of *lhx1-AS* that showed presence, reduction or absence of *pax8* expression at stage 20. *Lhx1-AS* 100 pg: reduced *pax8* expression was observed in 89%, absence in 4% and presence in 7% of the embryos (n = 28). *Lhx1-AS* 200 pg: reduced *pax8* expression was observed in 93% and presence in 7% of the embryos (n = 27). *Lhx1-AS* 300 pg: reduced of *pax8* expression was observed in 47% and absent in 53% of the embryos (n = 34). *Lhx1-AS* 400 pg: reduced *pax8* expression was observed in 50% and absent in 50% of the embryos (n = 42). *Lhx1-AS* 300 pg+*zflhx1* mRNA 25 pg: presence of *pax8* expression was observed in 51% of the embryos (n = 41).

Our *lhx1* over-expression results indicate this transcription factor has an important role in early specification of the renal progenitor cells. To further investigate the role of Lhx1 in pronephric kidney development, we injected embryos with *lhx1* N,N-diethylethylenediamine antisense oligonucleotide (*lhx1-AS*) [45]. Embryos were injected at the 8-cell stage (1xV2) with either *lhx1-AS* or a control N,N-diethylethylenediamine antisense oligonucleotide (*ctrl-AS*) [22]. We looked at the expression of the early pronephric marker *pax8* as an indication of kidney formation and observed a dose-dependent reduction of *pax8* expression on the injected side of stage 20 embryos (**Fig. S5**). As compared to uninjected embryos, 300 pg of *ctrl-AS* showed no effect on *pax8* expression (**Fig. 4A, 4B**). Injection of 300 pg of *lhx1-AS* caused either reduced or absent *pax8* expression in 100% of the embryos (**Fig. 4C, 4M**). Rescue experiments using zebrafish *lhx1* synthetic mRNA, insensitive to *lhx1-AS*, showed a marked rescue of the kidney field, which verified that the *lhx1-AS* phenotype is specific for depletion of *Xenopus lhx1* mRNA (**Fig. 4M**). The absence of the pronephric mesenchyme in *lhx1-AS* embryos indicates that expression of *lhx1* is essential for the early intermediate mesoderm patterning.

We analyzed expression of definitive kidney markers of the pronephric tubule using the antibodies 3G8 and 4A6. Injection of the *ctrl-AS* showed no differences with the uninjected embryos (**Fig. 4D, 4E, 4G, 4H**). The absence of 3G8 and 4A6 staining on the side of the embryos injected with 300 pg of *lhx1-AS* indicated a loss of differentiated cell types in the proximal and early distal regions of the pronephric kidney (76% and 91%, respectively) (**Fig. 4F, 4I**). We also confirmed the absence of the pronephric tubule by performing *in situ* hybridization for *β1-NaK-ATPase* expression. Pronephric transcripts of the *β*1 subunit appear at stage 26 and mark the onset of the maturation phase during pronephric kidney organogenesis [46]. As expected, we observed reduced *β1-NaK-ATPase* expression (72% of embryos) when *lhx1* expression was depleted (**Fig. 4L**). The lack of expression of these markers indicates an absence of differentiated pronephric kidney suggesting an essential role for Lhx1 in specification of the kidney field.

### *In vitro* explant culture and microarray analysis demonstrates *lhx1* expression is required for specification of the entire kidney field

The *lhx1-AS* depletion studies support the idea that *lhx1* is involved in driving specification of intermediate mesoderm into nephrogenic mesenchyme. *Lhx1* is initially expressed throughout the entire intermediate mesoderm (**Fig. S1**). Therefore, we wished to determine if the entire kidney field or specific sub-domains are affected by the absence of *lhx1*. Instead of using *lhx1-AS* targeted injections to study expression of kidney markers in whole embryos, one marker at a time, we performed a microarray analysis using an explant culture system. *Xenopus* tissue explants can be surgically isolated and cultured under specific conditions to be driven towards many distinct tissue types [47], [48]. Pronephric cell fates is induced by culturing isolated explants in the presence of Activin and RA (AcRA) [49], [50], [51]. Treatment of dissected explants from stage 8–9 embryos with 10 ng/ml Activin and 1×10^−4^ M RA can induce differentiation of the pluripotent ectoderm into pan-kidney tissue in approximately 90% of the treated explants [49]. For the *lhx1*-depletion experiments, both blastomeres of 2-cell embryos were injected with a total of 800 pg *lhx1-AS*. Explants were dissected and treated with AcRA and expression of *pax8* at stage 15 (based on timing of paired control whole embryos) was analyzed. We observed a lack of induction of *pax8* expression in *lhx1*-depleted explants under AcRA treatment conditions in which expression of this gene is normally induced (**Fig. S6A**). Based on this observation, microarray analysis was carried out to identify genes whose expression is affected by the absence of *lhx1*.

**Figure 5:**
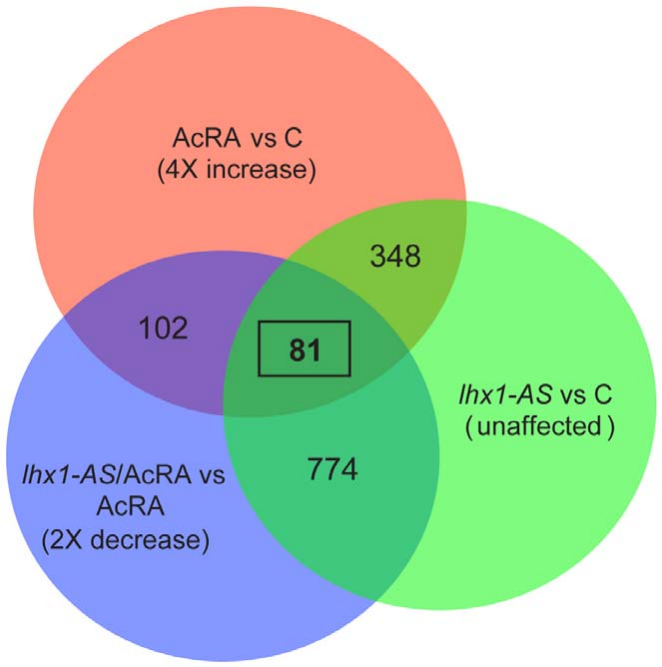
Microarray analysis of *lhx1*-depletion in organ culture. Venn diagram outlining the distribution of probe sets in the three organ culture treatments. Overlap between probe sets that showed a 4-fold or greater increase in expression in AcRA treated (AcRA) vs untreated (C) explants (pink), probe sets that presented a 2-fold or greater decrease in expression on *lhx1-AS*/AcRA vs AcRA explants (blue) and probe sets which expression showed less than a 0.5-fold increase/decrease in injected (*lhx1-AS*) vs C explants were considered unaffected (green).

Explants of embryos injected with 800 pg of *lhx1-AS* were dissected, treated with pronephric tissue inductive conditions (AcRA), and harvested between stages 13.5 and 14 (**Fig. S6B**). Explants from uninjected embryos treated with AcRA and untreated, as well as explants from *lhx1-AS* injected embryos untreated were also harvested. The microarray data was subjected to statistical analysis and pair-wise comparison of each sample was included. In order to produce a manageable data set of affected genes, the cutoff point for significant regulatory changes was set at a minimum of a 4-fold increase in gene expression of AcRA treated caps versus untreated control and 2-fold decrease or greater in gene expression of *lhx1-AS*/AcRA caps versus AcRA caps (**Fig. 5**). This statistical analysis resulted in a list of 81 probe sets for further study (**Fig. 5** **, S7**).

**Figure 6:**
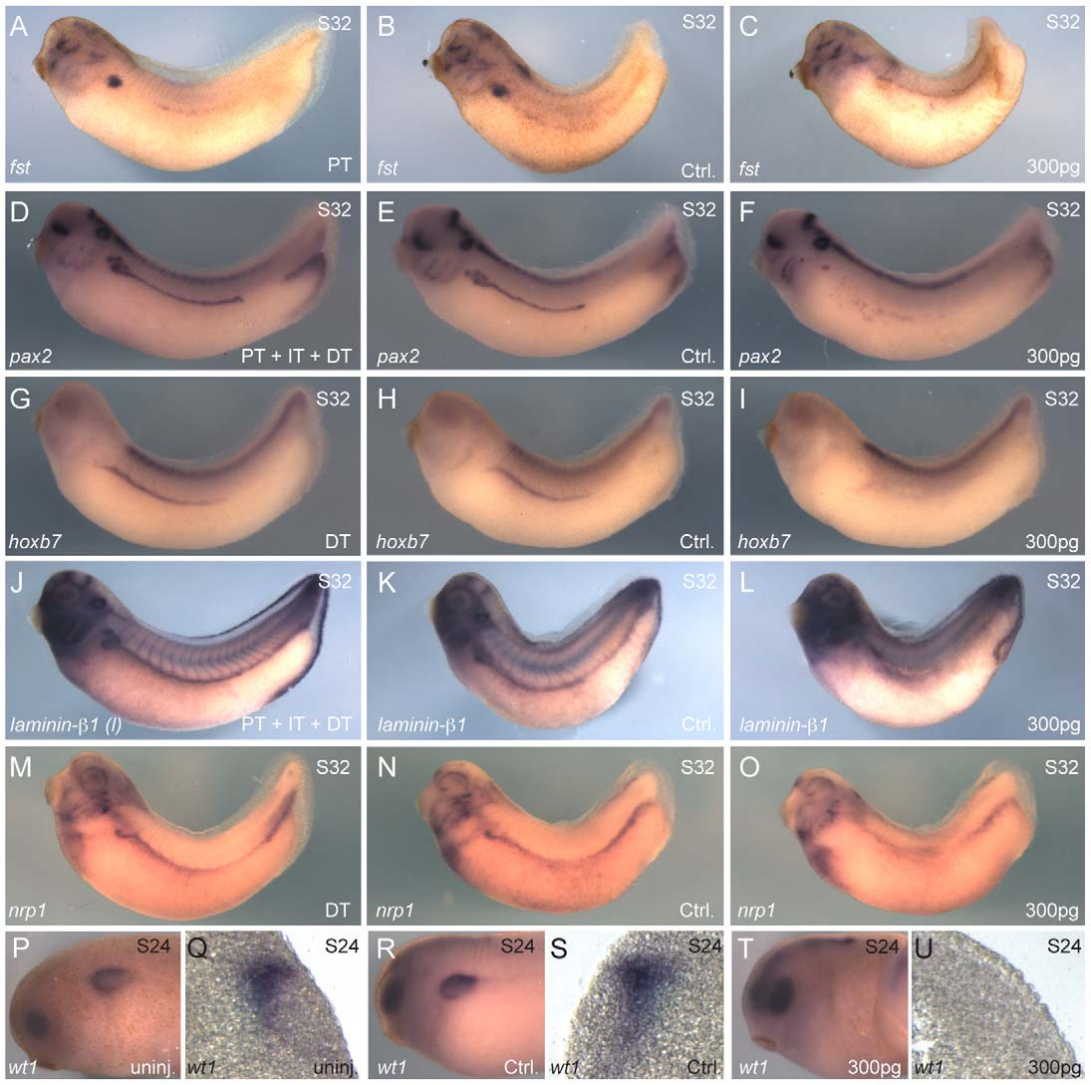
Absence of *lhx1* affects all the domains of the pronephric kidney. Embryos were injected (1xV2) with 300 pg of *lhx1-AS* at the 8-cell stage. (**A–O**) *In situ* hybridization of embryos at stage 32. (**A–C**) *In situ* hybridization for *follistatin (fst)*. (**A**) Uninjected embryo. (**B, C**) Control and injected sides of the same embryo are shown. (**C**) Reduced *fst* expression was observed in 57% of the embryos (n = 44). (**D–F**) *In situ* hybridization for *pax2*. (**D**) Uninjected embryo. (**E, F**) Control and injected sides of the same embryo are shown. (**F**) Reduced *pax2* expression was observed in 86% of the embryos (n = 32). (**G–I**) *In situ* hybridization for *hoxb7*. (**G**) Uninjected embryo. (**H, I**) Control and injected sides of the same embryo are shown. (**I**) Reduced *hoxb7* expression was observed in 92% of the embryos (n = 38). (**J–L**) *In situ* hybridization for *laminin-β1*. (**J**) Uninjected embryo. (**K, L**) Control and injected sides of the same embryo are shown. (**L**) Reduced *laminin-β1* expression was observed in 94% of the embryos (n = 32). (**M–O**) *In situ* hybridization for *neuropilin1 (nrp1)*. (**M**) Uninjected embryo. (**N, O**) Control and injected sides of the same embryo are shown. (**O**) Reduced *nrp1* expression was observed in 90% of the embryos (n = 40). (**P–U**) *In situ* hybridization of embryos at stage 24 for *wt1*. (**P, Q**) Uninjected embryo. (**R–U**) Control and injected sides of the same embryo are shown. (**T, U**) Reduced *wt1* expression was observed in 89% of the embryos (n = 37). (**Q, S, U**) Transverse sections of embryos in P and R/T, respectively. Arrows indicate plane of section in appropriate panels.

While AcRA treated explants induce pronephric tissue at a high frequency, the entire explant is not converted to kidney tissue and markers for neural tissue, epithelium and muscle are also expressed [49], [50], [51], [52]. For this reason, we considered it important to evaluate expression of our candidate genes using whole-mount *in situ* hybridization. Of the 81 hits found on the microarray (**Fig. 5**), we identified full-length cDNA or EST clones for 66 genes (**Fig. S7**) and performed *in situ* hybridization on embryos at stages 12.5, 15, 21 and 32. We identified a total of 17 genes with expression in the intermediate mesoderm at stage 15 (data not shown) and also later in the pronephric kidney at stage 32 (**Fig. 6A, 6D, 6J, 6M** **and Fig. S8**). Interestingly, we found genes from all domains of the pronephric tubule, lending support to the idea of Lhx1 being essential for the development of all kidney derivatives of the somatic layer of the intermediate mesoderm.

To confirm that absence of *lhx1* affects expression of the kidney genes found in the microarray assay, we injected 300 pg of *lhx1-AS* into 8-cell embryos (1xV2) and fixed them at stage 32 for *in situ* hybridization. We randomly selected markers to cover all regions of the pronephric kidney. The high stringency cutoff criteria and statistical analysis narrowly eliminated *hoxb7* from the final list. Since expression of this gene has been well characterized in the kidney of *Xenopus* and mice [53], [54], [55], we decided to include it and analyze the effect of loss of *lhx1* on the expression of this gene in whole embryos. Injected embryos showed a reduced expression of the proximal tubule markers, *follistatin* (**Fig. 6A–C** **)** and *pax2* (**Fig. 6D–F** **)** (57%, 86% respectively). When markers of distal and early distal tubule were analyzed in *lhx1-AS*-injected embryos, expression of *hoxb7* (**Fig. 6G–I** **)**, *laminin-b1* (**Fig. 6J–L** **)** and *neuropilin1* (**Fig. 6M–O** **)** was reduced in 92%, 94% and 90% of embryos, respectively. Since we did not find any glomerular markers among the 17 genes from the microarray, we decided to test whether both layers of the intermediate mesoderm [56] are affected by the absence of *lhx1* expression. For this purpose, we performed whole-mount *in situ* hybridization for *wt1*, a marker of the splanchnic intermediate mesoderm [57]. As compared to uninjected embryos (**Fig. 6P, 6Q**) and the control side (**Fig. 6R, 6S**), we observed reduced *wt1* expression in 89% of the *lhx1-AS* injected embryos (300 pg 1xV2) (**Fig. 6T, 6U**). These data suggest that both intermediate mesoderm derivatives of the kidney are affected by the absence of *lhx1*.

## Discussion

By studying the progression of intermediate mesoderm to pronephric kidney in *Xenopus*, we were able to readily gain access to embryos prior to the onset of kidney development. This allowed us to study the role of Lhx1 in specification of the nephric anlagen. By manipulating *lhx1* expression in *Xenopus* embryos we have established that Lhx1 function is required during the specification stage of pronephric kidney organogenesis. Expression of a constitutively-active form of Lhx1 results in the expansion of the kidney field and this ability is limited to the stages when specification of kidney progenitor cells is occurring [37], [52]. Our observation that Lhx1 plays a role in driving the intermediate mesoderm towards a renal progenitor cell population is in agreement with two current published datasets: (1) *lhx1* expression has been shown to be directly regulated by RA signaling, and potential RA responsive elements (RAREs) have been identified in the *lhx1* promoter [10]. Current dogma suggests that RA signaling is the primary step in establishing the intermediate mesoderm, thus putting *lhx1* directly downstream of this initial pattering event [10], [13], [58]. (2) *lhx1* perduration maintains the expression of early nephric markers and does not allow terminal differentiation of the kidney field to proceed [36].

Studies have identified a signal transduction pathway that drives specification of trunk mesoderm into distinct paraxial mesoderm and intermediate mesoderm fates [59]. Our results reveal the expansion of kidney field by over-expression of constitutively-active Ldb1-Lhx1 is associated with a decrease in paraxial mesoderm tissue. This result suggests that hyper-activation of Lhx1 is able to recruit cells from the paraxial mesoderm to adopt a nephric fate. These findings are in agreement with a report by Carroll *et al.*, which demonstrated ectopic kidney tissue induced by co-injection of *pax8* and *lhx1* is always found within the paraxial mesoderm or the intermediate mesoderm domains [25]. Temporally controlled activation of constitutively-active Ldb1-Lhx1 results in robust expansion of the kidney field at stages 15 and 18, during pronephric kidney specification. Notably, the kidney specification stage is the period when many known renal inductive factors are expressed [37]. Seeing as constitutively-active Ldb1-Lhx1 shows maximal effect during this inductive period, but not prior to this period, suggests that Lhx1 is possibly a competence factor necessary for establishing a broad kidney field, thereby making the field competent to respond to region specific renal inductive signals [60]. This hypothesis is supported by a recent report from Drews *et al.*, which demonstrated that Lhx1 does not have pronephric kidney inductive potential when over-expressed in a *Xenopus* explant system, but can influence the expression of multiple early nephrogenic factors [61].

Depletion of *lhx1* within the nephric anlagen using DEED-AS oligonucleotides resulted in a severe reduction or absence of the kidney tubule. Chan *et al.*, reported that injection of a dominant-negative form of Lhx1 into the C2 and C3 blastomeres of 32-cell embryos resulted in a disorganized and partial reduction of proximal tubules but more distal regions are not affected. Based on these results, they concluded that Lhx1 functions in proximal tubule morphogenesis [30]. Even though the C2/C3 blastomeres give rise to a portion of the kidney field, there is a substantial contribution from the surrounding blastomeres [62]. By injecting the V2 blastomere of 8-cell embryos, we targeted a larger segment of lineages that will give rise to the kidney [40]. Therefore, our observed reduction of marker gene expression from proximal through distal tubule cells is likely due to targeting more of the kidney field, demonstrating the importance of Lhx1 in specification of the entire pronephric tubule.

In order to identify all domains of the kidney affected by the absence of *lhx1* expression, we subjected *lhx1*-depleted kidney explants to microarray analysis. We found 81 probe sets that showed significant reduction of expression in kidney explants. Whole-mount *in situ* hybridization demonstrated that 17 of the clones are expressed in the kidney. Importantly, expression pattern analysis in whole embryos revealed that all segments of the pronephric kidney derived from the somatic layer of the intermediate mesoderm are altered when *lhx1* is depleted. Glomerular markers were not identified from the microarray experiments. It is possible glomerular markers were eliminated from our analysis due to a combination of low levels of expression of podocyte markers in the explants and the stringent statistical analysis of the microarray data. Additionally, a report by Carroll *et al.*, demonstrated that *lhx1* expression was present in the glomerular anlagen during specification stages, suggesting a possible role in establishing the glomus [63]. We evaluated expression of *wt1* in embryos injected with *lhx1-AS* and found expression of this gene to also be reduced. Therefore, Lhx1 function is essential for specification of both the somatic and splanchnic intermediate mesoderm layers. Previously reported microarray data analysis on mouse *lhx1* mutant metanephric mesenchyme, identified genes that are involved in patterning of the nephric vesicle [64], [65]. By using *Xenopus* as a model, we have been able to study the process of specification of the intermediate mesoderm, a stage of kidney development that is difficult to gain access to in mice, and that was not analyzed by the previous microarray studies. Taken together, our microarray analysis results indicate that Lhx1 is involved in specification of the entire kidney field. Further studies will be needed to identify additional factors that drive the process of regional kidney tissue induction in an Lhx1 competent kidney field.

## Methods

### Ethics Statement

This study was carried out in strict accordance with the recommendations in the Guide for the Care and Use of Laboratory Animals of the National Institutes of Health. The protocol (0812734A-2) was approved by the Institutional Animal Care and Use Committee of the University of Pittsburgh (Animal Welfare Assurance Number: A3187-01).

### *Xenopus* embryo manipulations and microinjections

*Xenopus laevis* embryos were obtained by artificial fertilization, maintained in 0.2× MMR and staged as previously described [5]. mRNA and/or *lhx1-AS* were co-injected with either fluorescein dextran or rhodamine dextran. Synthesis of LL-CA, LL-VP16 and zebrafish *lhx1* mRNAs were made using the SP6 mMessage mMachine kit (Ambion). For *lhx1* over-expression studies, 200–1200 pg of LL-CA or 50–300 pg of LL-VP16 were injected into one of the V2 blastomeres (1xV2) at the 8-cell stage. *Lhx1-AS* and *ctrl-AS* synthesis, sequence and specificity have been previously described [22], [45]. For *lhx1*-depletion experiments, 8-cell embryos were injected (1xV2) with 100–400 pg of *lhx1-AS* or *ctrl-AS*. For rescue experiments, 300 pg of *lhx1-AS* were coinjected with 25 pg of zebrafish *lhx1* mRNA into 8-cell embryos (1xV2). For temporally-controlled activation of Lhx1, the glucocorticoid receptor (GR) ligand-binding domain [66] was fused to the 3′ end of the LL-VP16 construct (LL-VP16-GR). 8-cell stage embryos were injected with 200 pg of LL-VP16-GR (1xV2). Injected embryos were treated with 10mM of dexamethasone (Dex) at indicated stages. For the cell proliferation studies, 200 pg of LL-VP16 were injected into 8-cell embryos (1xV2), and these embryos were subsequently treated with hydroxyurea and aphidicolin (HUA) from stage 10.5 as previously described [44].

### *In situ* hybridization and immunostaining

Whole-mount *in situ* hybridization was carried out as previously described [67]. The *pax8* construct, a gift from Tom Carroll, was linearized with the restriction enzyme *Not*I and transcribed with T7 to generate an antisense probe. The *myoD* and *scl* constructs were linearized with *Sal*I and transcribed with T7 to generate antisense probes. A *β1-NaK-ATPase* construct, gift from Oliver Wessely, was linearized with *EcoR*I and transcribed with T7 for an antisense probe. All other constructs used for probe synthesis were full-length cDNA or EST image clones obtained from Open Biosystems (http://openbiosystems.com/) and verified by sequencing. Whole-mount *in situ* hybridizations were developed using BM Purple AP Substrate (Roche). For whole-mount immunostaining, *Xenopus* embryos were fixed in Dent's (4∶1 methanol∶DMSO) for 4 hr at RT. After fixation embryos were washed with 100% methanol followed by rehydration. After incubation in blocking solution, embryos were transferred to a solution with 3G8 and/or 4A6 monoclonal antibody [42] followed by incubation with anti-mouse alkaline phosphatase IgG (Sigma) and developed using SIGMA*FAST* fast red tablets (Sigma). Monoclonal MZ15 and 12/101 antibodies were purchased from the Developmental Studies Hybridoma Bank. For double labeling experiments, embryos were fixed in MEMFA and processed for *in situ* hybridization followed by immunostaining. Monoclonal anti-phospho-Histone H3 antibody was purchased from Abcam. For transverse sections, the *in situ* hybridization processed embryos were embedded in low melting point agarose (Sigma, A0701) and then sectioned at 50 µm using a Vibratome (Leica VT1000 Series) [68].

### Explants assay

2-cell *Xenopus* embryos were injected at the animal pole with 800 pg of lhx1-AS and allowed to develop until stage 8–9, at which time the animal caps were dissected and cultured in 1× Danilchik's solution with 1×10^−4^ M RA and 10 ng/ml Activin when required [51]. Explants were cultured until the appropriate stage (described in the text) at which time they were assayed for pronephric kidney induction by RT-PCR or processed for microarray assays.

### RNA extraction and RT-PCR

Total RNA was extracted followed by cDNA synthesis and PCR as previously described [69]. The following PCR primers were used: *EF1a*forward primer,5′-CAGATTGGTGCTGGATATGC-3′; reverse primer, 5′-CACTGCCTTGATGACTCCTA-3′ [70], *Pax8* forward primer, 5′-CCAACAGCAGCATCAGATC-3′; reverse primer, 5′-CAATGACACCTGGCCGGATA-3′

### Microarray analysis

A total of 12 caps were pooled for each RNA preparation and the analysis was performed in triplicate. Total RNA was extracted from animal caps with the TRIzol (Invitrogen) isopropanol method, and purified with the RNeasy Kit (Qiagen). The biotin-labeled cDNA probe was generated using the Ovation Biotin system kit (NeuGen). Affymetrix GeneChip *Xenopus laevis* Genome Array (1st generation) was used for this assay. Hybridization, staining, and scanning were performed according to the instructions from Affymextrix. The signals were quantified, and normalized by GCOS (Affymetrix). Average signal value, *t*-test, and fold change were analyzed using JMP Statistical Discovery Software, (http://www.jmp.com/). Three replicates for each sample type were averaged, and probe sets showing differences with a *p* value smaller than 0.05 and fold value larger than 4-fold up or 2-fold down were considered as ‘change’ and selected for further study. The microarray data is MIAME compliant and the raw data has been deposited in the GEO database at NCBI (GSE24392).

## Supporting Information

Figure S1 ***Lhx1*** **and** ***pax8*** **expression in** ***Xenopus*** **embryos.** Expression of *lhx1* (**A–E**) and *pax8* (**F–J**) was visualized by *in situ* hybridization. The intermediate mesoderm (S12.5, S15) (**A, B, F, G**) and subsequent kidney field (S19.5, S21, S32) (**C–E, H–J**) are highlighted with black brackets.
(TIF)

Figure S2 **Dose-dependent expansion of the kidney field induced by LL-CA.** Embryos were injected (1xV2) with different doses of LL-CA mRNA at the 8-cell stage. (**A–C, E, F, H, I, K, L**) *In situ* hybridization of embryos at stage 20 for the early pronephric marker *pax8*. (**D, G, J, M**) Visualization of the injected side (asterisk) by the presence of fluorescein dextran. (**A**) Uninjected embryo. (**B–D**) Control and injected (200 pg) sides of the same embryo are shown. (**D**) Expansion of *pax8* expression was observed in 39% of the embryos (n = 31). (**E–G**) Control and injected (400 pg) sides of the same embryo are shown. (**F**) Expansion of *pax8* expression was observed in 42% of the embryos (n = 33). (**H–J**) Control and injected (800 pg) sides of the same embryo are shown. (**I**) Expansion of *pax8* expression was observed in 62% of the embryos (n = 34). (**K–M**) Control and injected (1200 pg) sides of the same embryo are shown. (**L**) Expansion of *pax8* expression was observed in 82% of the embryos (n = 33). (**N**) Bar graph with the percentage embryos injected with the different doses of LL-CA that showed expansion of *pax8* expression.
(TIF)

Figure S3 **Dose-dependent expansion of the kidney field induced by LL-VP16.** Embryos were injected (1xV2) with different doses of LL-VP16 mRNA at the 8-cell stage. (**A–C, E, F, H, I, K, L**) *In situ* hybridization of embryos at stage 20 for the early pronephric marker *pax8*. (**D, G, J, M**) Visualization of the injected side (asterisk) by the presence of fluorescein dextran. (**A**) Uninjected embryo. (**B–D**) Control and injected (50 pg) sides of the same embryo are shown. (**E–G**) Control and injected (100 pg) sides of the same embryo are shown. (**H–J**) Control and injected (200 pg) sides of the same embryo are shown. (**K–M**) Control and injected (300 pg) sides of the same embryo are shown. (**L**) Expansion of *pax8* expression was observed in 93% of the embryos (n = 27). Arrow indicates the misshapen kidney field.
(TIF)

Figure S4 **Analysis of secondary axis formation.** Embryos were injected (1xV2) with 200 pg of LL-VP16-GR mRNA at the 8-cell stage. (**A–L**) *In situ* hybridization for *pax8* of embryos at stage 31, followed by 12/101 whole-mount immunostaining. (**A, C, E, G, I, K**) Uninjected embryos. (**B, D, F, H, J, L**) Injected embryos. Activation of LL-VP16-GR was controlled by addition of dexamethasone (Dex) at specified stages. Dex was added to uninjected and injected embryos at: (**A, B**) stage 10; (**C, D**) stage 12.5; (**E, F**) stage 15; (**G, H**) stage 18; (**I, J**) stage 21; (**K, L**) stage 24.
(TIF)

Figure S5 **Dose-dependent reduction of the kidney field induced by** ***lhx1*** **depletion.** Embryos were injected (1xV2) with different doses of *lhx1-AS* at the 8-cell stage. (**A–C, E, F, H, I, K, L**) *In situ* hybridization of embryos at stage 20 for the early pronephric marker *pax8*. (**D, G, J, M**) Visualization of the injected side (asterisk) by the presence of fluorescein dextran. (**A**) Uninjected embryo. (**B–D**) Control and injected (100 pg) sides of the same embryo are shown. (**E–G**) Control and injected (200 pg) sides of the same embryo are shown. (**H–J**) Control and injected (300 pg) sides of the same embryo are shown. (**I**) Reduction of *pax8* expression was observed in 47% of the embryos and absence in 53% of the embryos (n = 34). (**K–M**) Control and injected (400 pg) sides of the same embryo areshown.
(TIF)

Figure S6 **Depletion of** ***lhx1*** **in explants for microarray analysis.** (**A**) RT-PCR analysis on lhx1-AS-injected animal caps (AC) treated with Activin and retinoic acid (AcRA). Induction of *pax8* expression in animal caps at stage 15 by AcRA is inhibited by injection of 800 pg of *lhx1-AS*. WE: whole embryos. RT-PCR controls: -RNA, -RT and -cDNA. *EF1α* was used as loading control. (**B**) Schematic of the procedure followed for the microarray analysis of animal caps. 2-cell embryos were injected in both blastomeres with a total of 800 pg of *lhx1-AS*. Embryos injected with *lhx1-AS* and uninjected were cultured until blastula stage (stage 8/9) when animal caps were dissected and cultured until stage 13.5/14 in the presence or absence of AcRA in the media. C: untreated animal caps.
(TIF)

Figure S7 **Probe sets showing up-regulated expression in the AcRA treated caps and down-regulated expression in injected** ***lhx1-AS*** **/AcRA caps.** Those genes in bold were found to be expressed in the kidney. Fold increase, refers to the relative increase in gene expression in AcRA vs untreated explants. Fold decrease, refers to the relative decrease in gene expression in *lhx1-AS*/AcRA vs AcRA explants. Abbreviation: WMIH, whole-mount *in situ* hybridization.
(PDF)

Figure S8 **Expression of kidney genes identified from the microarray analysis.** Whole-mount *in situ* hybridization of stage 32 embryos was performed. Expression was found in different domains of the pronephric kidney: proximal tubule (PT), early distal tubule (EDT), distal tubule (DT), and connecting tubule (CT). (**A, B, H–L**) Genes with expression in the PT. (**C–L**) Genes with expression in the EDT. (**E–L**) Genes with expression in the DT. (**M**) Gene with expression in the CT.
(TIF)

## References

[1] Bouchard M, Souabni A, Mandler M, Neubuser A, Busslinger M (2002). Nephric lineage specification by Pax2 and Pax8.. Genes Dev.

[2] James RG, Schultheiss TM (2003). Patterning of the avian intermediate mesoderm by lateral plate and axial tissues.. Dev Biol.

[3] Mauch TJ, Yang G, Wright M, Smith D, Schoenwolf GC (2000). Signals from trunk paraxial mesoderm induce pronephros formation in chick intermediate mesoderm.. Dev Biol.

[4] Dressler GR (2006). The cellular basis of kidney development.. Annu Rev Cell Dev Biol.

[5] Nieuwkoop PD, Faber J (1994). Normal table of Xenopus Laevis.

[6] Herzlinger D (1995). Inductive interactions during kidney development.. Semin Nephrol.

[7] Serluca FC, Fishman MC (2001). Pre-pattern in the pronephric kidney field of zebrafish.. Development.

[8] Guillaume R, Bressan M, Herzlinger D (2009). Paraxial mesoderm contributes stromal cells to the developing kidney.. Dev Biol.

[9] Seufert DW, Brennan HC, DeGuire J, Jones EA, Vize PD (1999). Developmental basis of pronephric defects in Xenopus body plan phenotypes.. Dev Biol.

[10] Cartry J, Nichane M, Ribes V, Colas A, Riou JF (2006). Retinoic acid signalling is required for specification of pronephric cell fate.. Dev Biol.

[11] Mendelsohn C, Lohnes D, Decimo D, Lufkin T, LeMeur M (1994). Function of the retinoic acid receptors (RARs) during development (II). Multiple abnormalities at various stages of organogenesis in RAR double mutants.. Development.

[12] Wingert RA, Davidson AJ (2008). The zebrafish pronephros: a model to study nephron segmentation.. Kidney Int.

[13] Osafune K, Nishinakamura R, Komazaki S, Asashima M (2002). In vitro induction of the pronephric duct in Xenopus explants.. Dev Growth Differ.

[14] James RG, Schultheiss TM (2005). Bmp signaling promotes intermediate mesoderm gene expression in a dose-dependent, cell-autonomous and translation-dependent manner.. Dev Biol.

[15] Dawid IB, Breen JJ, Toyama R (1998). LIM domains: multiple roles as adapters and functional modifiers in protein interactions.. Trends Genet.

[16] Dawid IB, Toyama R, Taira M (1995). LIM domain proteins.. C R Acad Sci III.

[17] Agulnick AD, Taira M, Breen JJ, Tanaka T, Dawid IB (1996). Interactions of the LIM-domain-binding factor Ldb1 with LIM homeodomain proteins.. Nature.

[18] Breen JJ, Agulnick AD, Westphal H, Dawid IB (1998). Interactions between LIM domains and the LIM domain-binding protein Ldb1.. J Biol Chem.

[19] Taira M, Jamrich M, Good PJ, Dawid IB (1992). The LIM domain-containing homeo box gene Xlim-1 is expressed specifically in the organizer region of Xenopus gastrula embryos.. Genes Dev.

[20] Harland R, Gerhart J (1997). Formation and function of Spemann's organizer.. Annu Rev Cell Dev Biol.

[21] Lemaire P, Kodjabachian L (1996). The vertebrate organizer: structure and molecules.. Trends Genet.

[22] Hukriede NA, Tsang TE, Habas R, Khoo PL, Steiner K (2003). Conserved requirement of Lim1 function for cell movements during gastrulation.. Dev Cell.

[23] Kodjabachian L, Karavanov AA, Hikasa H, Hukriede NA, Aoki T (2001). A study of Xlim1 function in the Spemann-Mangold organizer.. Int J Dev Biol.

[24] Barnes JD, Crosby JL, Jones CM, Wright CV, Hogan BL (1994). Embryonic expression of Lim-1, the mouse homolog of Xenopus Xlim-1, suggests a role in lateral mesoderm differentiation and neurogenesis.. Dev Biol.

[25] Carroll TJ, Vize PD (1999). Synergism between Pax-8 and lim-1 in embryonic kidney development.. Dev Biol.

[26] Swanhart LM, Takahashi N, Jackson RL, Gibson GA, Watkins SC (2010). Characterization of an lhx1a transgenic reporter in zebrafish.. Int J Dev Biol.

[27] Toyama R, Dawid IB (1997). lim6, a novel LIM homeobox gene in the zebrafish: comparison of its expression pattern with lim1.. Dev Dyn.

[28] Tsang TE, Shawlot W, Kinder SJ, Kobayashi A, Kwan KM (2000). Lim1 activity is required for intermediate mesoderm differentiation in the mouse embryo.. Dev Biol.

[29] Taira M, Otani H, Jamrich M, Dawid IB (1994). Expression of the LIM class homeobox gene Xlim-1 in pronephros and CNS cell lineages of Xenopus embryos is affected by retinoic acid and exogastrulation.. Development.

[30] Chan TC, Takahashi S, Asashima M (2000). A role for Xlim-1 in pronephros development in Xenopus laevis.. Dev Biol.

[31] Diep CQ, Ma D, Deo RC, Holm TM, Naylor RW (2011). Identification of adult nephron progenitors capable of kidney regeneration in zebrafish.. Nature.

[32] Fujii T, Pichel JG, Taira M, Toyama R, Dawid IB (1994). Expression patterns of the murine LIM class homeobox gene lim1 in the developing brain and excretory system.. Dev Dyn.

[33] Shawlot W, Behringer RR (1995). Requirement for Lim1 in head-organizer function.. Nature.

[34] Kobayashi A, Kwan KM, Carroll TJ, McMahon AP, Mendelsohn CL (2005). Distinct and sequential tissue-specific activities of the LIM-class homeobox gene Lim1 for tubular morphogenesis during kidney development.. Development.

[35] Pedersen A, Skjong C, Shawlot W (2005). Lim 1 is required for nephric duct extension and ureteric bud morphogenesis.. Dev Biol.

[36] Agrawal R, Tran U, Wessely O (2009). The miR-30 miRNA family regulates Xenopus pronephros development and targets the transcription factor Xlim1/Lhx1.. Development.

[37] Brandli AW (1999). Towards a molecular anatomy of the Xenopus pronephric kidney.. Int J Dev Biol.

[38] Brennan HC, Nijjar S, Jones EA (1998). The specification of the pronephric tubules and duct in Xenopus laevis.. Mech Dev.

[39] Wu G, Bohn S, Ryffel GU (2004). The HNF1beta transcription factor has several domains involved in nephrogenesis and partially rescues Pax8/lim1-induced kidney malformations.. Eur J Biochem.

[40] Huang S, Johnson KE, Wang HZ (1998). Blastomeres show differential fate changes in 8-cell Xenopus laevis embryos that are rotated 90 degrees before first cleavage.. Dev Growth Differ.

[41] Raciti D, Reggiani L, Geffers L, Jiang Q, Bacchion F (2008). Organization of the pronephric kidney revealed by large-scale gene expression mapping.. Genome Biol.

[42] Vize PD, Jones EA, Pfister R (1995). Development of the Xenopus pronephric system.. Dev Biol.

[43] Smith JC, Watt FM (1985). Biochemical specificity of Xenopus notochord.. Differentiation.

[44] Harris WA, Hartenstein V (1991). Neuronal determination without cell division in Xenopus embryos.. Neuron.

[45] Dagle JM, Weeks DL (2001). Oligonucleotide-based strategies to reduce gene expression.. Differentiation.

[46] Eid SR, Brandli AW (2001). Xenopus Na,K-ATPase: primary sequence of the beta2 subunit and in situ localization of alpha1, beta1, and gamma expression during pronephric kidney development.. Differentiation.

[47] Ariizumi T, Asashima M (2001). In vitro induction systems for analyses of amphibian organogenesis and body patterning.. Int J Dev Biol.

[48] Green JB, Smith JC (1990). Graded changes in dose of a Xenopus activin A homologue elicit stepwise transitions in embryonic cell fate.. Nature.

[49] Chan TC, Ariizumi T, Asashima M (1999). A model system for organ engineering: transplantation of in vitro induced embryonic kidney.. Naturwissenschaften.

[50] Moriya N, Komazaki S, Takahashi S, Yokota C, Asashima M (2000). In vitro pancreas formation from Xenopus ectoderm treated with activin and retinoic acid.. Dev Growth Differ.

[51] Moriya N, Uchiyama H, Asashima M (1993). Induction of pronephric tubules by activin and retinoic acid in presumptive ectoderm of Xenopus laevis.. Development, Growth and Differenciation.

[52] Brennan HC, Nijjar S, Jones EA (1999). The specification and growth factor inducibility of the pronephric glomus in Xenopus laevis.. Development.

[53] Godsave S, Dekker EJ, Holling T, Pannese M, Boncinelli E (1994). Expression patterns of Hoxb genes in the Xenopus embryo suggest roles in anteroposterior specification of the hindbrain and in dorsoventral patterning of the mesoderm.. Dev Biol.

[54] Plaisier E, Ribes D, Ronco P, Rossert J (2005). Identification of two candidate collecting duct cell-specific cis-acting elements in the Hoxb-7 promoter region.. Biochim Biophys Acta.

[55] Vogels R, Charite J, de Graaff W, Deschamps J (1993). Proximal cis-acting elements cooperate to set Hoxb-7 (Hox-2.3) expression boundaries in transgenic mice.. Development.

[56] Vize PD, Seufert DW, Carroll TJ, Wallingford JB (1997). Model systems for the study of kidney development: use of the pronephros in the analysis of organ induction and patterning.. Dev Biol.

[57] Carroll TJ, Vize PD (1996). Wilms' tumor suppressor gene is involved in the development of disparate kidney forms: evidence from expression in the Xenopus pronephros.. Dev Dyn.

[58] Wingert RA, Selleck R, Yu J, Song HD, Chen Z (2007). The cdx genes and retinoic acid control the positioning and segmentation of the zebrafish pronephros.. PLoS Genet.

[59] Wilm B, James RG, Schultheiss TM, Hogan BL (2004). The forkhead genes, Foxc1 and Foxc2, regulate paraxial versus intermediate mesoderm cell fate.. Dev Biol.

[60] Slack JM (1993). Embryonic induction.. Mech Dev.

[61] Drews C, Senkel S, Ryffel GU (2011). The nephrogenic potential of the transcription factors osr1, osr2, hnf1b, lhx1 and pax8 assessed in Xenopus animal caps.. BMC Dev Biol.

[62] Dale L, Slack JM (1987). Fate map for the 32-cell stage of Xenopus laevis.. Development.

[63] Carroll TJ, Wallingford JB, Vize PD (1999). Dynamic patterns of gene expression in the developing pronephros of Xenopus laevis.. Dev Genet.

[64] Chen YT, Kobayashi A, Kwan KM, Johnson RL, Behringer RR (2006). Gene expression profiles in developing nephrons using Lim1 metanephric mesenchyme-specific conditional mutant mice.. BMC Nephrol.

[65] Potter SS, Hartman HA, Kwan KM, Behringer RR, Patterson LT (2007). Laser capture-microarray analysis of Lim1 mutant kidney development.. Genesis.

[66] Kolm PJ, Sive HL (1995). Efficient hormone-inducible protein function in Xenopus laevis.. Dev Biol.

[67] Gawantka V, Pollet N, Delius H, Vingron M, Pfister R (1998). Gene expression screening in Xenopus identifies molecular pathways, predicts gene function and provides a global view of embryonic patterning.. Mech Dev.

[68] Cho GS, Choi SC, Park EC, Han JK (2011). Role of Tbx2 in defining the territory of the pronephric nephron.. Development.

[69] Barnett MW, Old RW, Jones EA (1998). Neural induction and patterning by fibroblast growth factor, notochord and somite tissue in Xenopus.. Dev Growth Differ.

[70] Mohun TJ, Taylor MV, Garrett N, Gurdon JB (1989). The CArG promoter sequence is necessary for muscle-specific transcription of the cardiac actin gene in Xenopus embryos.. EMBO J.

