# Supplementary figures and images for: Lhx1 Is Required for Specification of the Renal Progenitor Cell Field
Source: PLoS One. 2011 Apr 15;6(4):e18858. doi: 10.1371/journal.pone.0018858 (PMC3078140; doi:10.1371/journal.pone.0018858)

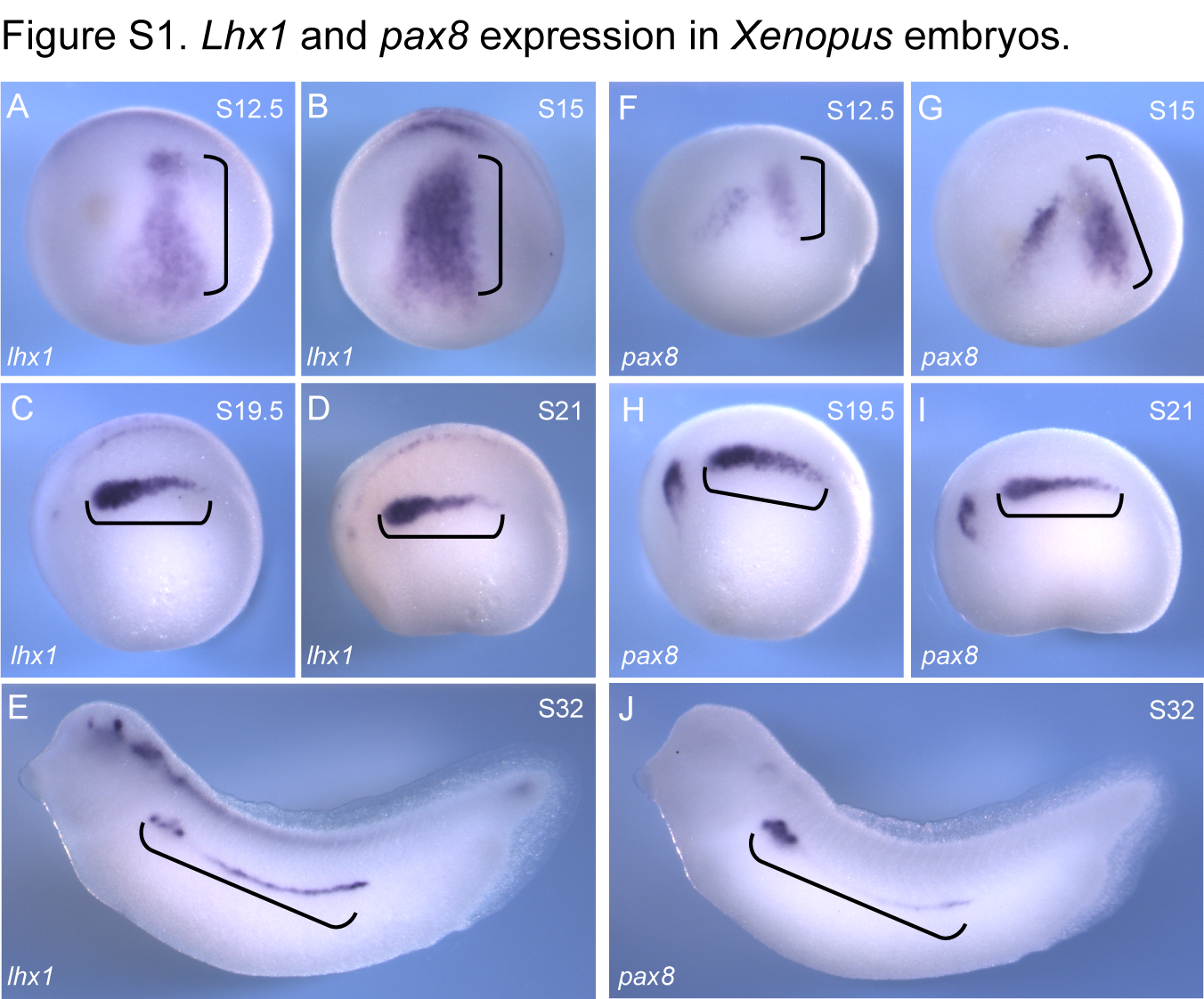

Supplement: Figure S1 — Lhx1 and pax8 expression in Xenopus embryos. Expression of lhx1 (A–E) and pax8 (F–J) was visualized by in situ hybridization. The intermediate mesoderm (S12.5, S15) (A, B, F, G) and subsequent kidney field (S19.5, S21, S32) (C–E, H–J) are highlighted with black brackets. (TIF) [file pone.0018858.s001.tif]

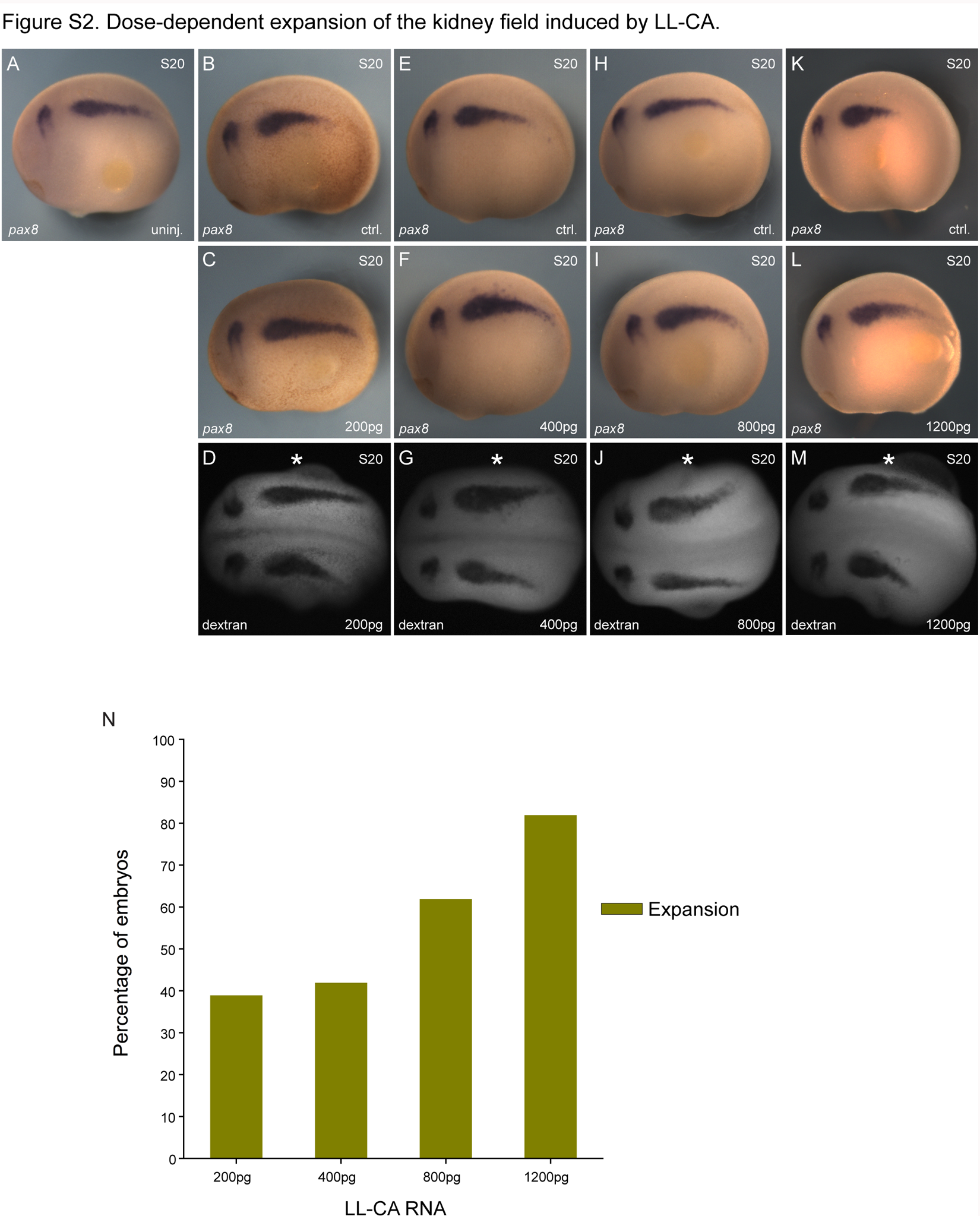

Supplement: Figure S2 — Dose-dependent expansion of the kidney field induced by LL-CA. Embryos were injected (1xV2) with different doses of LL-CA mRNA at the 8-cell stage. (A–C, E, F, H, I, K, L) In situ hybridization of embryos at stage 20 for the early pronephric marker pax8. (D, G, J, M) Visualization of the injected side (asterisk) by the presence of fluorescein dextran. (A) Uninjected embryo. (B–D) Control and injected (200 pg) sides of the same embryo are shown. (D) Expansion of pax8 expression was observed in 39% of the embryos (n = 31). (E–G) Control and injected (400 pg) sides of the same embryo are shown. (F) Expansion of pax8 expression was observed in 42% of the embryos (n = 33). (H–J) Control and injected (800 pg) sides of the same embryo are shown. (I) Expansion of pax8 expression was observed in 62% of the embryos (n = 34). (K–M) Control and injected (1200 pg) sides of the same embryo are shown. (L) Expansion of pax8 expression was observed in 82% of the embryos (n = 33). (N) Bar graph with the percentage embryos injected with the different doses of LL-CA that showed expansion of pax8 expression. (TIF) [file pone.0018858.s002.tif]

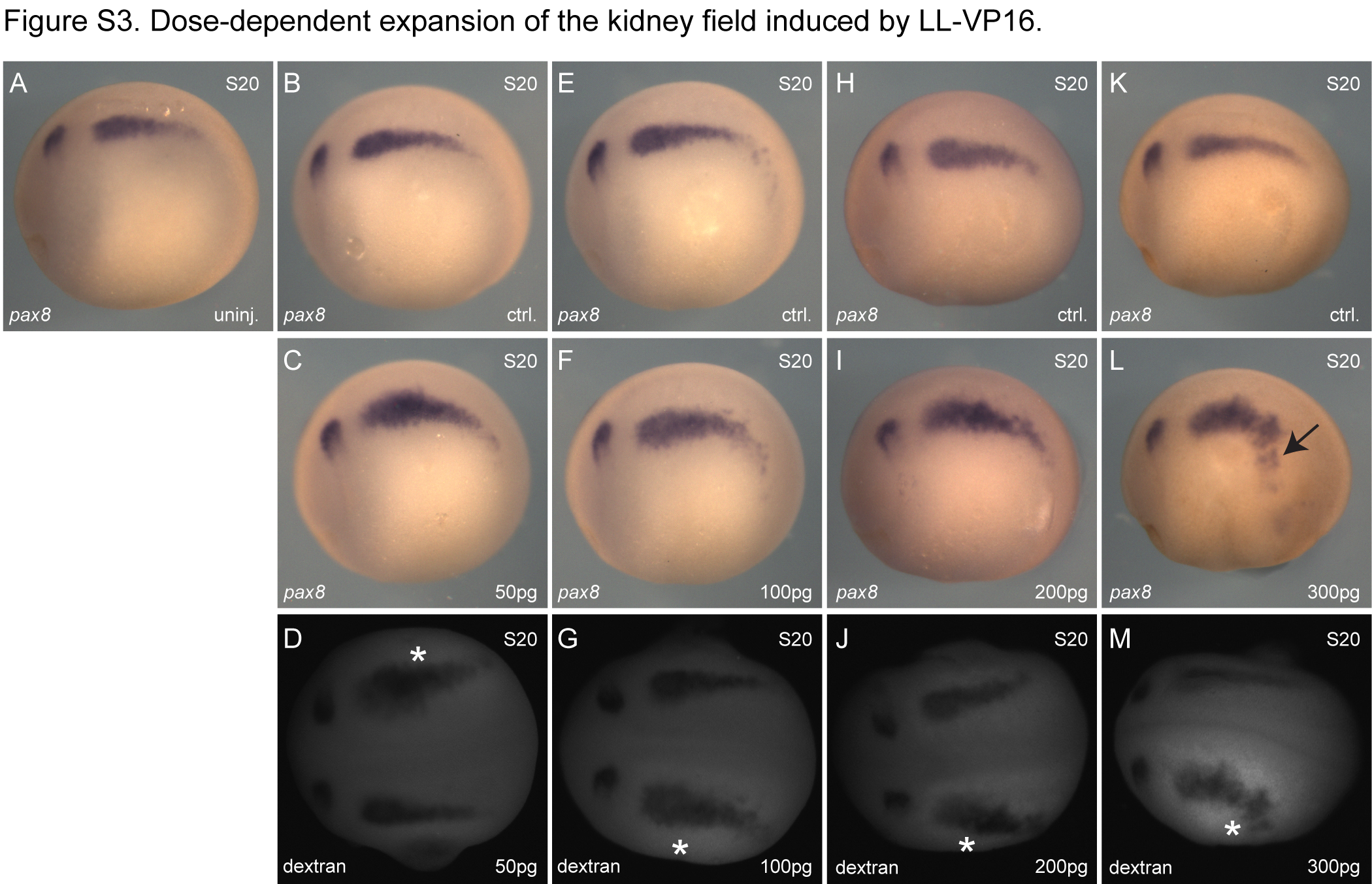

Supplement: Figure S3 — Dose-dependent expansion of the kidney field induced by LL-VP16. Embryos were injected (1xV2) with different doses of LL-VP16 mRNA at the 8-cell stage. (A–C, E, F, H, I, K, L) In situ hybridization of embryos at stage 20 for the early pronephric marker pax8. (D, G, J, M) Visualization of the injected side (asterisk) by the presence of fluorescein dextran. (A) Uninjected embryo. (B–D) Control and injected (50 pg) sides of the same embryo are shown. (E–G) Control and injected (100 pg) sides of the same embryo are shown. (H–J) Control and injected (200 pg) sides of the same embryo are shown. (K–M) Control and injected (300 pg) sides of the same embryo are shown. (L) Expansion of pax8 expression was observed in 93% of the embryos (n = 27). Arrow indicates the misshapen kidney field. (TIF) [file pone.0018858.s003.tif]

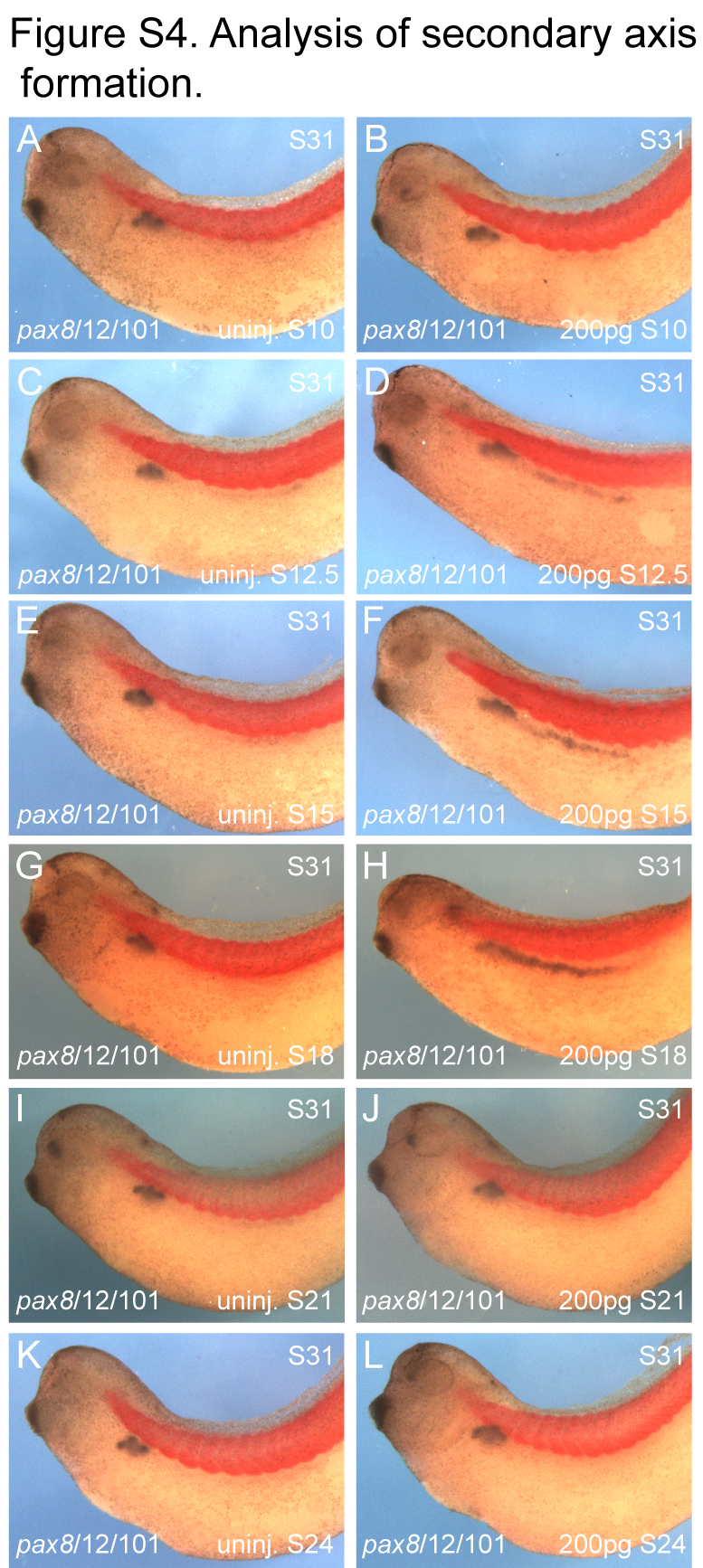

Supplement: Figure S4 — Analysis of secondary axis formation. Embryos were injected (1xV2) with 200 pg of LL-VP16-GR mRNA at the 8-cell stage. (A–L) In situ hybridization for pax8 of embryos at stage 31, followed by 12/101 whole-mount immunostaining. (A, C, E, G, I, K) Uninjected embryos. (B, D, F, H, J, L) Injected embryos. Activation of LL-VP16-GR was controlled by addition of dexamethasone (Dex) at specified stages. Dex was added to uninjected and injected embryos at: (A, B) stage 10; (C, D) stage 12.5; (E, F) stage 15; (G, H) stage 18; (I, J) stage 21; (K, L) stage 24. (TIF) [file pone.0018858.s004.tif]

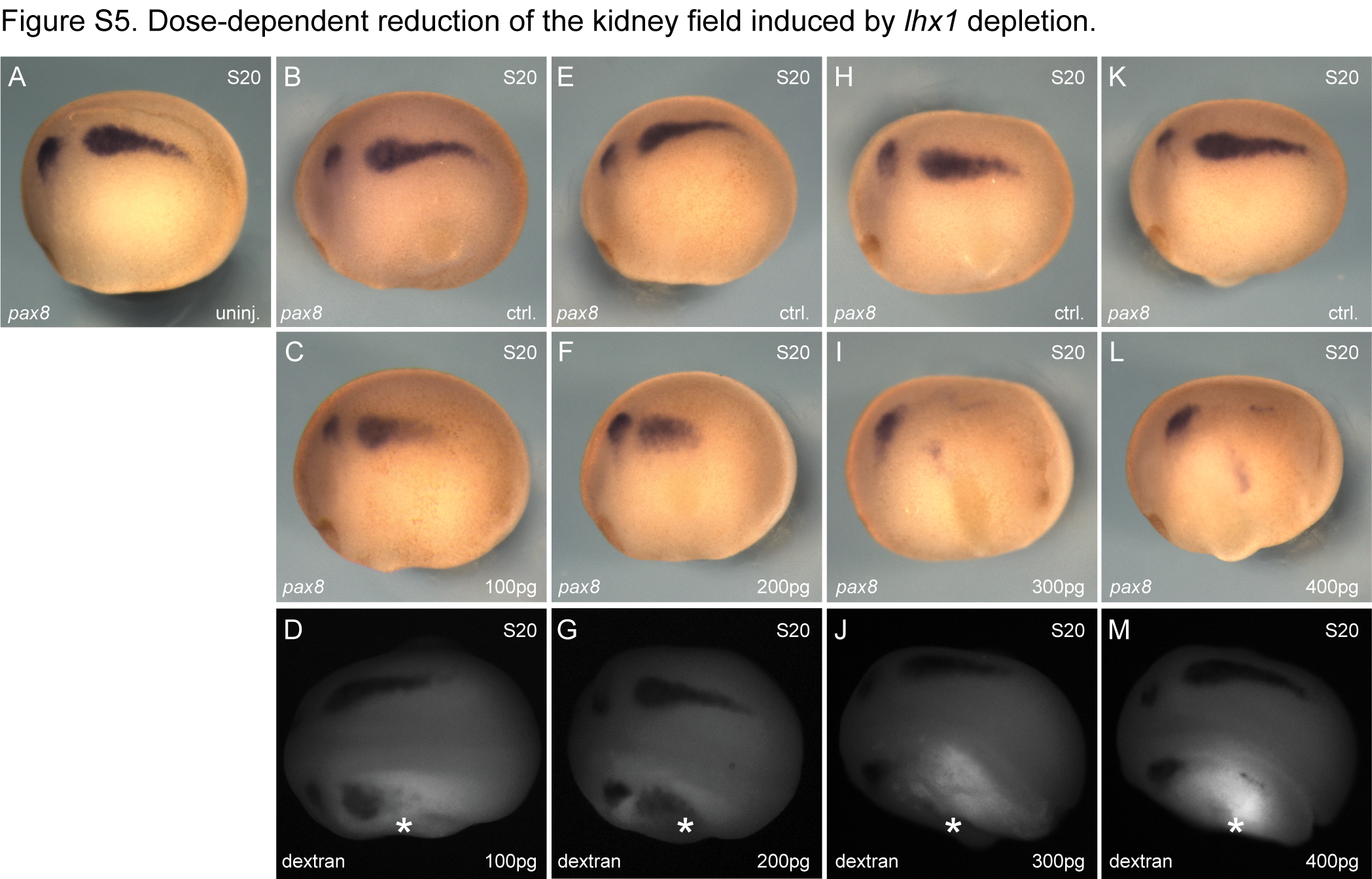

Supplement: Figure S5 — Dose-dependent reduction of the kidney field induced by lhx1 depletion. Embryos were injected (1xV2) with different doses of lhx1-AS at the 8-cell stage. (A–C, E, F, H, I, K, L) In situ hybridization of embryos at stage 20 for the early pronephric marker pax8. (D, G, J, M) Visualization of the injected side (asterisk) by the presence of fluorescein dextran. (A) Uninjected embryo. (B–D) Control and injected (100 pg) sides of the same embryo are shown. (E–G) Control and injected (200 pg) sides of the same embryo are shown. (H–J) Control and injected (300 pg) sides of the same embryo are shown. (I) Reduction of pax8 expression was observed in 47% of the embryos and absence in 53% of the embryos (n = 34). (K–M) Control and injected (400 pg) sides of the same embryo areshown. (TIF) [file pone.0018858.s005.tif]

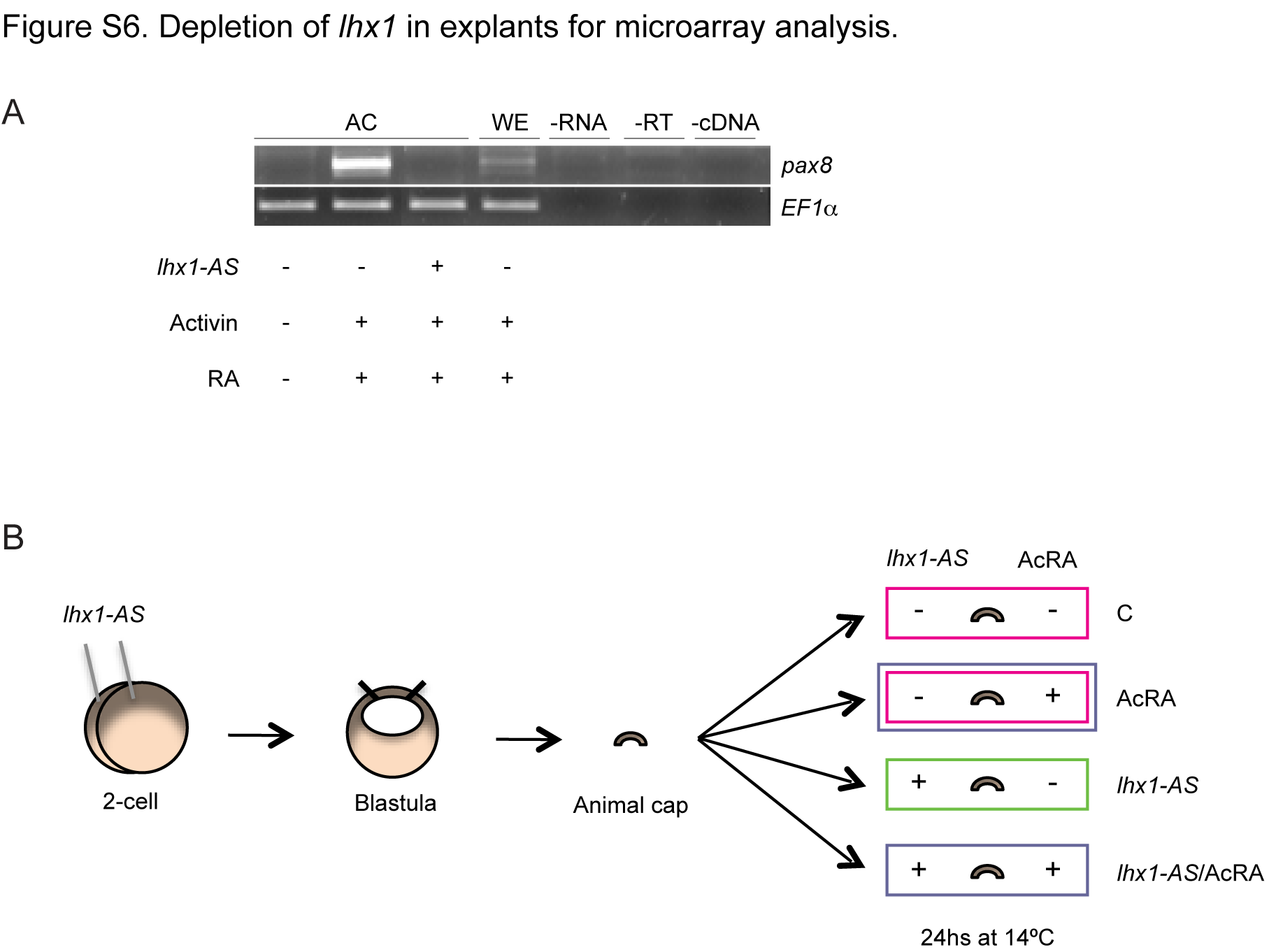

Supplement: Figure S6 — Depletion of lhx1 in explants for microarray analysis. (A) RT-PCR analysis on lhx1-AS-injected animal caps (AC) treated with Activin and retinoic acid (AcRA). Induction of pax8 expression in animal caps at stage 15 by AcRA is inhibited by injection of 800 pg of lhx1-AS. WE: whole embryos. RT-PCR controls: -RNA, -RT and -cDNA. EF1α was used as loading control. (B) Schematic of the procedure followed for the microarray analysis of animal caps. 2-cell embryos were injected in both blastomeres with a total of 800 pg of lhx1-AS. Embryos injected with lhx1-AS and uninjected were cultured until blastula stage (stage 8/9) when animal caps were dissected and cultured until stage 13.5/14 in the presence or absence of AcRA in the media. C: untreated animal caps. (TIF) [file pone.0018858.s006.tif]

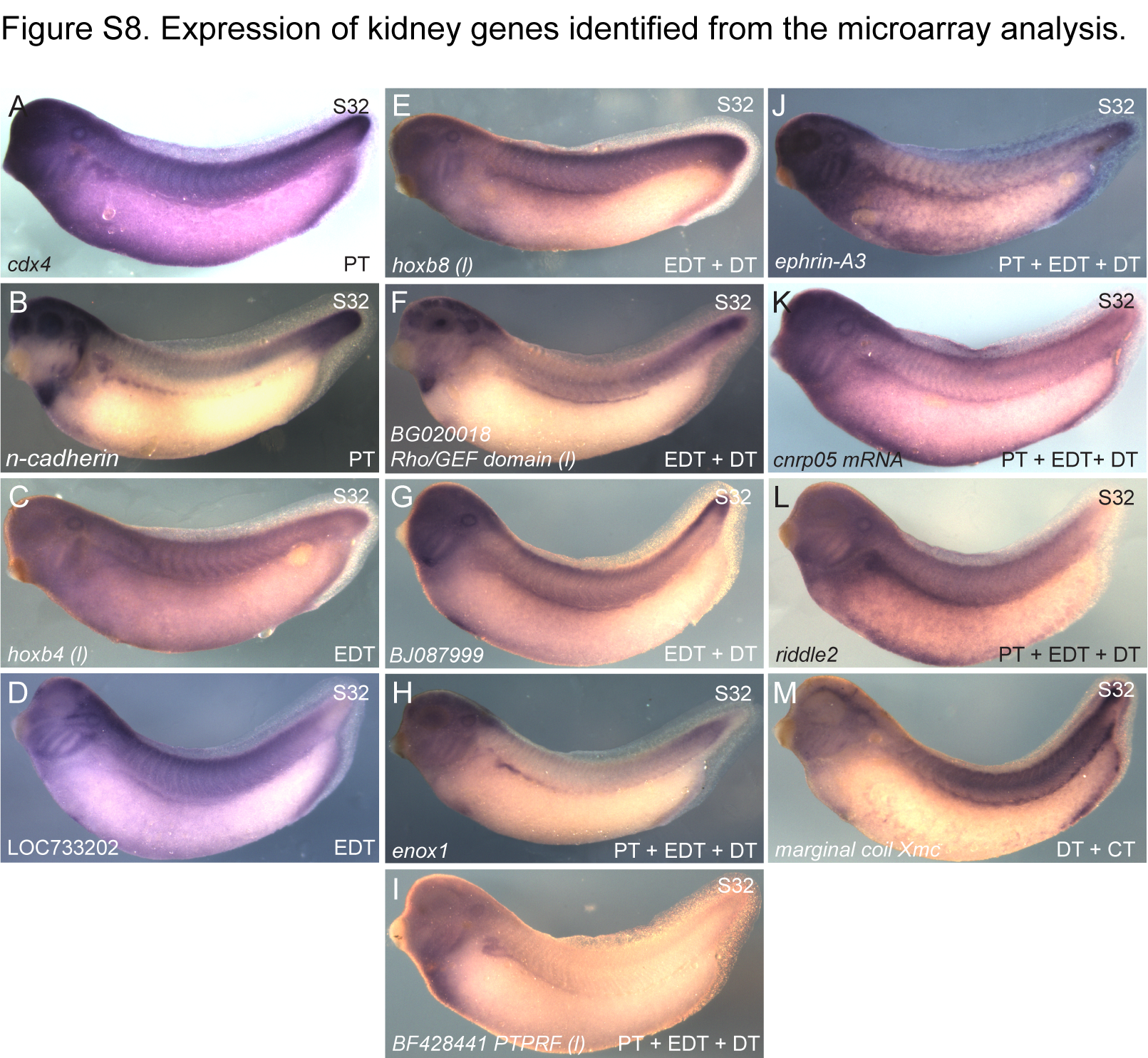

Supplement: Figure S8 — Expression of kidney genes identified from the microarray analysis. Whole-mount in situ hybridization of stage 32 embryos was performed. Expression was found in different domains of the pronephric kidney: proximal tubule (PT), early distal tubule (EDT), distal tubule (DT), and connecting tubule (CT). (A, B, H–L) Genes with expression in the PT. (C–L) Genes with expression in the EDT. (E–L) Genes with expression in the DT. (M) Gene with expression in the CT. (TIF) [file pone.0018858.s008.tif]
