# Supplementary material for: Lhx1 Is Required for Specification of the Renal Progenitor Cell Field
Source: PLoS One. 2011 Apr 15;6(4):e18858. doi: 10.1371/journal.pone.0018858 (PMC3078140; doi:10.1371/journal.pone.0018858)
Supplement: Figure S7 — Probe sets showing up-regulated expression in the AcRA treated caps and down-regulated expression in injected lhx1-AS /AcRA caps. Those genes in bold were found to be expressed in the kidney. Fold increase, refers to the relative increase in gene expression in AcRA vs untreated explants. Fold decrease, refers to the relative decrease in gene expression in lhx1-AS/AcRA vs AcRA explants. Abbreviation: WMIH, whole-mount in situ hybridization. (PDF) [file pone.0018858.s007.pdf]

Gene Name/GenBank ID	*Fold Increase*	*Fold decresase*	*WMIH*
Protocadherin PCNS	207	4	+
Teashirt zinc finger homeobox 3	162	4	+
**Caudal type homeo box 4**	133	6	+
BM179670	127	5	+
Wingless-type MMTV integration site family, m	117	4	+
Lapl06 mRNA	99	19	+
MGC84445	90	5	+
Xcad1	86	6	+
**MGC131119 (Homeobox B4 like)**	80	4	+
**Homeobox B8 like**	75	5	+
**BJ087999**	70	13	+
Paired box 3	48	4	+
AW460602	44	2	+
Thylacine 1	34	2	+
**Marginal coil Xmc**	26	8	+
MGC131003	25	3	+
Factor I C3b/C4b inactivator (Cfi)	24	3	+
Microfibrillar-associated protein 2	23	7	+
BF048418	22	4	+
**BG885063 (Neuropilin 1 like)**	22	14	+
BJ091281	21	4	-
AFFX-Xl-a1Act-3_s_at	20	30	-
Axin-related protein	20	3	+
Wingless-type MMTV integration site family, m	19	27	-
**cDNA clone IMAGE:6949617 (hypothetica**	18	5	+
cDNA clone MGC:98998 IMAGE:5513759	17	3	+
**Follistatin**	17	4	+
BJ077645	16	4	+
BI350356	13	2	+
BG346022	12	6	-
Kringle containing transmembrane protein 2	12	5	+
BJ056491	11	7	+
XMeis1-3	11	6	+
BJ044843	11	7	-
LOC446245	11	7	+
Secreted protein, acidic, cysteine-rich	10	14	+
Myosin light chain	10	4	-
BG579880	10	3	+
CDNA clone MGC:82481	9	7	+
**ecto-NOX disulfide-thiol exchanger 1**	8	5	+
BJ083371	8	10	+
**Riddle 2**	8	3	+
BG161264	8	5	-
Collagen, type II, alpha 1	8	8	+
LOC734178	8	6	+
Wingless-type MMTV integration site family, m	8	11	+
**Homeobox B7**	8	2	+
BG730739	8	8	-

Fibrillin 1	7	3	+
BJ047004	7	24	-
BJ078265	7	3	+
**N-cadherin 5' non-coding region**	7	4	+
BJ079589	7	5	+
Retinoic acid receptor, alpha	6	3	+
MGC81784 protein	6	3	+
BJ075680	6	7	+
BE490942	6	2	-
Homeobox A3	6	3	+
**BF428441 (PTPRF binding protein 2 like)**	6	7	+
BI447733	6	2	-
**BG020018 (Rho/GEF domain like)**	5	2	+
MGC53495	5	4	+
AW766736	5	16	-
**BM260404 (Cnpr05 mRNA)**	5	3	+
**BQ400974 (Laminin beta-1 chain precurs**	5	2	+
Zinc finger protein GLI4	5	3	+
Fibroblast growth factor receptor 1	5	2	+
**Ephrin-A3**	5	3	+
BJ085082	5	11	-
LOC733321	5	7	+
CB564510	5	7	-
BG347424	5	13	-
CB756533	5	4	-
MGC84737 protein	4	2	+
BG345987	4	3	-
XMeis1-2	4	3	+
**Paired box gene 2**	4	3	+
LIN-28 like	4	2	+
Meso05 mRNA	4	3	+
SRY (sex determining region Y)-box 12	4	3	+
Spondin-2	4	7	+
Syntaxin binding protein 1	4	20	+
